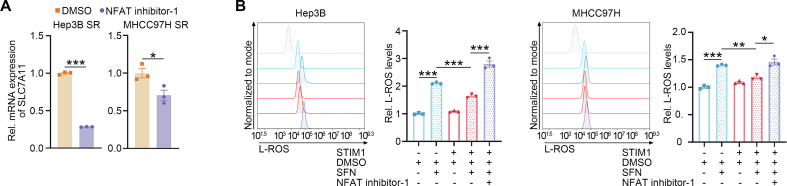# STIM1 promotes acquired resistance to sorafenib by attenuating ferroptosis in hepatocellular carcinoma

**DOI:** 10.1016/j.gendis.2024.101281

**Published:** 2024-03-28

**Authors:** Ran Ren, Yu Chen, Yu Zhou, Luyao Shen, Yang Chen, Juan Lei, Jingchun Wang, Xudong Liu, Nan Zhang, Dongqin Zhou, Huakan Zhao, Yongsheng Li

**Affiliations:** a Chongqing University Cancer Hospital, School of Medicine, Chongqing University, Chongqing 400044, China; b Department of Medical Oncology, Chongqing University Cancer Hospital, Chongqing 400030, China; c The Second School of Medicine, Wenzhou Medical University, Wenzhou, Zhejiang 325027, China; d Department of Gastroenterology, Xinqiao Hospital, Army Medical University, Chongqing 400037, China

**Keywords:** Ferroptosis, Hepatocellular carcinoma, SLC7A11, Sorafenib resistance, STIM1

## Abstract

Dysregulated calcium (Ca^2+^) signaling pathways are associated with tumor cell death and drug resistance. In non-excitable cells, such as hepatocellular carcinoma (HCC) cells, the primary pathway for Ca^2+^ influx is through stromal interaction molecule 1 (STIM1)-mediated store-operated calcium entry (SOCE). Previous studies have demonstrated the involvement of STIM1-mediated SOCE in processes such as genesis, metastasis, and stem cell self-renewal of HCC. However, it remains unclear whether STIM1-mediated SOCE plays a role in developing acquired resistance to sorafenib in HCC patients. In this study, we established acquired sorafenib-resistant (SR) HCC cell lines by intermittently exposing them to increasing concentrations of sorafenib. Our results showed higher levels of STIM1 and stronger SOCE in SR cells compared with parental cells. Deleting STIM1 significantly enhanced sensitivity to sorafenib in SR cells, while overexpressing STIM1 promoted SR by activating SOCE. Mechanistically, STIM1 increased the transcription of SLC7A11 through the SOCE-CaN-NFAT pathway. Subsequently, up-regulated SLC7A11 increased glutathione synthesis, resulting in ferroptosis insensitivity and SR. Furthermore, combining the SOCE inhibitor SKF96365 with sorafenib significantly improved the sensitivity of SR cells to sorafenib both *in vitro* and *in vivo*. These findings suggest a potential strategy to overcome acquired resistance to sorafenib in HCC cells.

## Introduction

Liver cancer is a significant global health challenge, ranking sixth in terms of incidence and third in terms of mortality. Hepatocellular carcinoma (HCC) accounts for about 90% of liver cancer cases.^1^ Sorafenib, the first multi-target tyrosine kinase inhibitor approved by the U.S. Food and Drug Administration for systemic treatment of advanced HCC patients, is a crucial component of targeted therapy for HCC and is supported by robust clinical data.^2^ While newer 1st-line systemic agents have been developed for the treatment of advanced HCC, a novel strategy to treat HCC has emerged the combination of sorafenib with immunotherapy. Sorafenib, a multi-kinase inhibitor with anti-angiogenic effects, has the potential to enhance anti-tumor immune responses by synergistically regulating the vasculature and the immune microenvironment of the tumor.^3^^,^^4^ However, patients with advanced HCC often develop acquired resistance to sorafenib within 6 months,^5^ posing a major obstacle to improving clinical efficacy.

Calcium (Ca^2+^), an important intracellular second messenger, is closely associated with various cellular activities, including cell proliferation, apoptosis, immunity, and drug resistance.^6^^,^^7^ The primary pathway for Ca^2+^ influx in non-excitable cells, particularly tumor cells, is through store-operated Ca^2+^ entry (SOCE), which is mediated by stromal interaction molecule 1 (STIM1).^8^ Our previous studies revealed that STIM1 promotes the growth of HCC in a hypoxic environment, accelerates metabolic reprogramming in HCC, leading to enhanced invasion and metastasis, and enhances self-renewal of liver cancer stem cells.^9^, ^10^, ^11^ Furthermore, studies have demonstrated that STIM1 inhibits apoptosis in most tumors, except prostate cancer^12^^,^^13^ and promotes autophagy in certain tumor types.^14^ However, the involvement of STIM1-mediated SOCE in the development of acquired resistance to sorafenib in HCC patients remains unknown.

Ferroptosis, a form of programmed cell death that relies on iron and is characterized by the accumulation of lipid reactive oxygen species (L-ROS), was first discovered by Dixon et al in 2012.^15^ Growing evidence suggests that ferroptosis plays a crucial role in tumor drug resistance, particularly in sorafenib resistance (SR), and targeting ferroptosis may provide new opportunities to overcome drug resistance in different types of cancer.^16^ Currently, three main pathways have been identified in ferroptosis: amino acid metabolism, lipid metabolism, and iron metabolism. The amino acid metabolic pathway, which is primarily regulated by glutathione (GSH) metabolism, plays a central role in the regulation of ferroptosis. The synthesis of GSH relies on the cystine/glutamate antiporter, also known as the Xc^−^ system, located in the cell membrane. This antiporter consists of two subunits, solute carrier family 7 member 11 (SLC7A11) and SLC3A2. Additionally, GSH is converted to glutathione peroxidase 4 (GPX4) to counteract L-ROS and attenuate ferroptosis.

In this study, we aimed to investigate the expression of STIM1 in HCC cells that have developed resistance to sorafenib. We hypothesized that the impact of STIM1-mediated calcium signaling on ferroptosis might play a role in the acquired resistance to sorafenib in HCC. Gain- and loss-of-function studies were conducted to investigate the role of STIM1 in sorafenib sensitivity and ferroptosis, along with the underlying mechanism. Furthermore, the effect of combining the SOCE inhibitor SKF96365 and sorafenib was evaluated both *in vitro* and *in vivo*. Taken together, this study elucidates the mechanism of sorafenib resistance and identifies STIM1 as a potential target for overcoming this resistance in HCC cells.

## Material and methods

### Human samples

Seven pairs of HCC tissues were obtained from patients at the Chongqing University Cancer Hospital (Chongqing, China). The use of clinical specimens in this study was approved by the Ethics Committee of Chongqing University Cancer Hospital and informed written consent was obtained from all participants. The information of HCC patients involved in this study is shown in Table S1.

### Cell lines

Hep3B (RRID: CVCL_0326), MHCC97H (RRID: CVCL_4972), and HEK293T (RRID: CVCL_0063) cells were obtained from the GuanDao Biological Engineering Co., Ltd. (Shanghai, China). All human cell lines have been authenticated using STR profiling within the last three years, and they were tested for mycoplasma. The cells were maintained following the manufacturer's instructions.

### Establishment of sorafenib-resistant Hep3B and MHCC97H cell lines

Parental Hep3B and MHCC97H cells were exposed to increasing concentrations of sorafenib in two-week cycles over more than 6 months. Subsequently, these cells were maintained with high concentrations of sorafenib.

### Cell viability and lactate dehydrogenase release assay

Cell viability was assessed using the Cell Counting Kit-8 (CCK-8) (BioGround, Chongqing, China). Hep3B cells were seeded at a density of 1 × 10^5^ cells/well, while MHCC97H cells were seeded at a density of 5 × 10^4^ cells/well, in a 96-well plate. Following overnight incubation at 37 °C, the original medium was replaced with a new medium containing the drug. After 72 h of further incubation at 37 °C, 1 × CCK-8 solution was added and the absorbance at 450 nm was measured using a microtiter plate reader after 2 h of incubation at 37 °C. For the lactate dehydrogenase release assay, the CytoTox 96 Non-Radioactive Cytotoxicity Assay Kit (Promega, Madison, USA) was used. The experimental procedure was similar to that of the CCK-8 experiment. After 72 h of incubation with the drug, 100 μL of supernatant was collected and transferred to a new 96-well plate. Then, 100 μL of CytoTox working solution was added and incubated for 30 min. The absorbance at 490 nm was measured using a microtiter plate reader.

### Real-time quantitative PCR

Total RNAs were extracted using the Eastep Super Total RNA Extraction Kit (Promega, Madison, USA). Reverse transcription was performed using the PrimeScript RT Reagent Kit (Takara, Osaka, Japan). Real-time quantitative PCR was conducted using the SYBR qPCR Master Mix (Takara, Osaka, Japan). The primer sequences can be found in Table S2. The expression of the target gene was normalized to the ACTIN level, and the fold change was calculated using the 2^−ΔΔCt^ method.

### Western blotting

Protein extraction was performed using RIPA lysis buffer supplemented with phenylmethyl sulfonyl fluoride (Beyotime, Beijing, China). For nuclear protein isolation, cells were lysed using the Nuclear and Cytoplasmic Protein Extraction Kit (Beyotime, Beijing, China), following the manufacturer's instructions. The protein concentrations were determined using a BCA Protein Assay Kit (Epizyme, Shanghai, China). Subsequently, proteins from each experimental group were separated by 4%–12% Bis-tris SDS-PAGE gels (Genscript, Nanjing, China) and transferred onto a PVDF membrane (Millipore, Boston, USA) for subsequent detection. The membrane was then incubated at 4 °C overnight with primary antibodies (1:1000) targeting the respective proteins of interest. Following this, the membrane was washed three times with Tris-Buffered Saline Tween-20 and incubated with secondary antibodies (1:5000) at room temperature for 1 h. Antigens were visualized using a chemiluminescence assay with Pierce ECL Western (Invitrogen, Carlsbad, USA). The specific antibodies utilized in this study are listed in Table S3.

### Calcium imaging

Cells were seeded into a 24-well plate coated with a poly-d-lysine round coverslip. The buffer solution used for the experiment contained the following concentrations (in mM): 140 NaCl, 5 KCl, 2 CaCl_2_, 1 MgCl_2_·6H_2_O, 10 Glucose, and 10 HEPES, with a pH of 7.4. To prepare the Ca^2+^-free buffer solution, CaCl_2_ was replaced with equimolar amounts of MgCl_2_·6H_2_O, and 0.5 mM EGTA was added. After washing the cells three times with the Ca^2+^-free buffer, a 5 μM intracellular Ca^2+^ fluorescent indicator called Fura-2 AM (Invitrogen, Carlsbad, USA) diluted with Ca^2+^-free buffer was added. The cells were then incubated at 37 °C in the dark for 1 h. Subsequently, the cells were washed three times with Ca^2+^-free buffer and further incubated for 30 min to ensure complete de-esterification. Measurements of intracellular Ca^2+^ concentration in single cells were performed using an inverted fluorescence microscope (Nikon, Japan). Images were collected at 6-s intervals. At 120 s, the buffer solution was switched to a Ca^2+^-free buffer containing 200 μM cyclopiazonic acid (MCE, Monmouth Junction, USA), and at 360 s, it was switched to a Ca^2+^-containing buffer with cyclopiazonic acid. The assay was terminated at 600 s. The results were expressed as the ratios of fluorescence signals measured at 340 nm to that at 380 nm during a response, divided by the ratio obtained under resting conditions.

### CRISPR/Cas9 targeted deletion of STIM1

To knock down the STIM1 gene, we designed single-guided RNA (sgRNA) sequences (Forward: 5′-CACCGCATCATCGTCCATCAGTTTG-3′; Reverse: 5′-AAACCAAACTGATGGACGATGATGC-3′) specifically targeting the human STIM1 gene. These targeting sequences were then cloned into the lentiCRISPR v2 vector, which also carries the green fluorescence protein (Addgene, Watertown, USA). The Hep3B SR and MHCC97H SR cells were transfected with the plasmid using Lipofectamine 3000 (Invitrogen, Carlsbad, USA) for a duration of 24 h. Following transfection, the cells were selected with G418 (400 μg/mL) (Beyotime, Beijing, China) for 2 weeks. Subsequently, the green fluorescence protein-positive cells were sorted using flow cytometry to establish monoclonal cells. The deletion of STIM1 in each monoclonal cell line was further confirmed through western blotting.

### Lentiviral and siRNA

Recombinant lentiviral plasmids containing human STIM1 or NFATc2 (nuclear factor of activated T cells 2) were obtained from GeneCopoeia. To produce lentivirus, HEK293T cells were used with the Lenti-Pac HIV Expression Packaging Kit (GeneCopoeia, Rockville, USA). After 48 h of transfection, supernatants were collected by centrifugation at 2000 *g* for 10 min Hep3B and MHCC97H cells were infected with lentivirus for 72 h and then selected with puromycin (2 μg/mL) (Beyotime, Beijing, China) for 2 weeks. The overexpression of STIM1 was confirmed through real-time quantitative PCR and western blotting analysis. siRNAs targeting human NFATc1, NFATc2, NFATc3, and NFATc4 were purchased from RiboBio (Guangzhou, China). Cells were transfected using Lipofectamine 3000 (Invitrogen, Carlsbad, USA) following the manufacturer's instructions.

### RNA sequencing

The total RNA of tumor cells was isolated using the Trizol Reagent (Takara, Osaka, Japan). Subsequently, mRNA with a polyA structure in the total RNA was enriched using oligo-magnetic beads. The RNA was then fragmented to approximately 300 bp length by ion interruption. The first strand of cDNA was synthesized using base random primers and reverse transcriptase, and the second strand of cDNA was synthesized using the RNA as a template. After constructing the library, PCR amplification was performed to enrich the library fragments. The library was assessed for quality using the Agilent 2100 Bioanalyzer (Santa Clara, USA). Then, based on the desired amount of data and the effective concentration of the library, libraries with different index sequences were mixed proportionally. Single-stranded libraries were formed through base denaturation. Following RNA extraction, purification, and library construction, the libraries underwent paired-end sequencing using next-generation sequencing on the Illumina sequencing platform. The raw next-generation sequencing data underwent detailed quality control and filtering, as described in Tables S4–7.

### Colony formation assays

Cells were seeded into a 6-well plate and incubated for three days. After this period, the original medium was replaced with a new medium containing the specific drug. Following a 72-h incubation, the cells were washed twice with phosphate-buffered saline and then fixed with 4% paraformaldehyde for 10 min. Subsequently, they were washed twice with distilled water for 2 min each time. Cell clones were then stained with crystal violet staining solution (Beyotime, Beijing, China) for 10 min and thoroughly washed with distilled water before being photographed.

### GSH quantification and malondialdehyde (MDA) assay

Total GSH and MDA levels were measured using either the GSSG/GSH Quantification Kit II or the MDA Assay Kit (DojinDo, Kumamoto, Japan). Hep3B cells were seeded at a density of 1 × 10^7^ cells/well, while MHCC97H cells were seeded at a density of 5 × 10^6^ cells/well in a 10 cm dish. The cells were lysed following the instruction manual, and the working solution was added. The GSH levels were determined by measuring the absorbance at 405 nm or 415 nm using a microtiter plate reader. Alternatively, the MDA levels were assessed by detecting the fluorescence intensity at Ex: 540 nm and Em: 590 nm using a microtiter plate reader.

### L-ROS assay

L-ROS was detected using Fluorescent Dye BODIPY 581/591C11 or BODIPY 665/676 (Invitrogen, Carlsbad, USA). In brief, a total of 4 × 10^5^ Hep3B cells and 2 × 10^5^ MHCC97H cells were seeded into a 12-well plate. After overnight incubation, the cells were treated with either DMSO as a control or sorafenib, SKF96365, and ferrostatin-1 for 72 h. BODIPY 581/591 C11 or BODIPY 665/676 were prepared as a 5 μM working solution using RPMI 1640 medium. Each well was washed twice with phosphate-buffered saline, followed by the addition of 500 μL of the working solution, and then incubated at 37 °C for 1 h. The cells were subsequently collected, washed, and resuspended, and the samples were analyzed using Agilent Novocyte Advanteon (Santa Clara, USA) flow cytometry and Novoexpress software.

### Transmission electron microscopy

For Hep3B cells, 1 × 10^7^ cells/well were seeded into a 10 cm dish, while for MHCC97H cells, 5 × 10^6^ cells/well were seeded into the same dish. The cells were then collected and fixed by slowly adding 2.5% glutaraldehyde (MCE, Monmouth Junction, USA). After that, the cell samples were dehydrated with acetone and permeated with epoxy resin and acetone. The samples were then embedded into silica gel moulds, polymerized, and sectioned. Prior to observation under transmission electron microscopy, the cell samples were stained with 1% uranyl acetate and basic lead citrate.

### Iron quantification

Intracellular levels of Fe^2+^ were measured using a FerroOrange fluorescent probe (DojinDo, Kumamoto, Japan). For this assay, cells were treated with an HBSS buffer solution containing 1 μm/L FerroOrange. After incubation at 37 °C for 30 min, the intracellular Fe^2+^ levels were visualized under a fluorescence microscope and analyzed using ImageJ.

### Immunofluorescence staining

The frozen sections were initially fixed with 4% paraformaldehyde (Beyotime, Beijing, China) for 15 min. They were then washed three times with phosphate-buffered saline and incubated with permeabilization buffer (Beyotime, Beijing, China) for 40 min. After 2 h of blocking, the primary antibody (1:100) was added and incubated at 4 °C overnight. Following three washes with phosphate-buffered saline Tween-20, the secondary antibody (1:500) was added. Finally, the sections were stained with an Antifade Reagent with DAPI (CST, Boston, USA) and observed using Leica fluorescence confocal microscopy. The antibodies used in immunofluorescence staining are listed in Table S3. To quantify the levels of STIM1 and SLC7A11, we used ImageJ (National Institutes of Health, USA) to measure the mean fluorescence intensity of each field of view. For STIM1, we defined “STIM1 low” as a mean fluorescence intensity below 30, and “STIM1 high” as a mean fluorescence intensity above 30.

### Hematoxylin-eosin staining and immunohistochemistry staining

The tumor tissue was fixed in 4 % paraformaldehyde for a minimum of 24 h. It was then dehydrated using isopropyl alcohol and the alcohol was subsequently removed with xylene. The dehydrated samples were embedded in paraffin and stained with hematoxylin and eosin for staining. For immunohistochemistry staining, the sections were incubated in citrate buffer at 95 °C for 45 min to extract antigens. Prior to incubating with the primary antibody at 4 °C overnight, the sections were incubated with 5% goat serum at 37 °C for 30 min. After three washes, the sections were incubated with the secondary antibody at room temperature for 60 min. Finally, the sections were incubated with a 3,3′-diaminobenzidine solution and stained with hematoxylin. The staining results were captured using an Olympus digital sectioning workstation and analyzed with ImageJ. The antibodies used in immunohistochemistry staining are listed in Table S3.

### Animal experiments

Four-week-old male NOD.CB17-Prkdcscid/l2rgtm1/Bcgen (B-NDG) mice were obtained from Biocytogen Pharmaceuticals (Beijing, China) Co., Ltd. They were kept in pathogen-free conditions prior to the experiment. Xenograft tumor models were constructed by mixing the cell suspension with Matrigel (Corning, New York, USA) in a 1:1 ratio, and injecting the mixture subcutaneously into the right axilla of B-NDG mice. Tumor growth was regularly monitored, and intraperitoneal injections of the drug were initiated when the tumor volume reached 100 mm^3^ and given every other day. When the tumor volume was reduced to 50 mm^3^, the mice were euthanized to obtain the tumor tissue for subsequent immunofluorescence or immunohistochemistry staining experiments.

### Co-immunoprecipitation

The cross-linking product of the target protein was captured using the Protein A/G Magnetic Beads (Bimake, Bimake, USA) according to the instruction manual. Cells were harvested in protease inhibitor cocktail lysis buffer (CST, Boston, USA). The cell lysates were mixed with either the primary antibody or normal mouse IgG and incubated at room temperature for 2 h. After that, the washed magnetic beads were added to the cell lysate and incubated overnight at 4 °C. The beads were washed three times with phosphate-buffered saline. Finally, the samples were eluted into an SDS sampling buffer and analyzed using western blotting. The antibodies used in co-immunoprecipitation are listed in Table S3.

### Chromatin immunoprecipitation

Chromatin immunoprecipitation experiments were conducted following the protocol outlined in the SimpleChIP Plus Enzymatic Chromatin IP Kit (Magnetic Beads) instruction manual (CST, Boston, USA, Cat: #9005). To initiate DNA crosslinking, cells were incubated with 37% formaldehyde at room temperature for 10 min. The reaction was then terminated by adding glycine, and the cells were collected. Nuclei were prepared using a specific buffer and fragmented through sonication, and the resulting supernatant was collected for chromatin digestion and immunoprecipitation. Subsequently, the chromatin was eluted from the Antibody/Protein G Magnetic Beads and de-crosslinked, followed by DNA purification. Finally, the DNA was quantified using PCR. The antibodies and primers used in chromatin immunoprecipitation are listed in Tables S3 and 8, respectively.

### Luciferase reporter assay

Cells were seeded into 12-well plates and transfected with negative control or lentiviral plasmids containing human NFATc2 and luciferase reporter vectors. After 24 h, the cells were lysed and tested using a dual-luciferase reporter assay kit (Promega, Madison, USA) following the manufacturer's instructions.

### Statistical analysis

Statistical analysis was performed using GraphPad Prism 8.0.2 software, and the results were presented as mean ± standard error of the mean. Two-tailed Student's *t*-test was conducted to determine statistical significance. A *P* value of less than 0.05 was considered statistically significant. Each experiment was repeated at least three times, and representative images of western blotting assays, immunofluorescence staining, immunohistochemical staining, transmission electron microscope, and DNA agarose gel blot were provided.

## Results

### The expression of STIM1 is up-regulated in HCC cell models with acquired SR

To investigate the association between STIM1 and acquired SR in HCC cells, we initially developed SR HCC cell lines. This was done by subjecting parental Hep3B and MHCC97H cells to gradually increasing concentrations of sorafenib for approximately 6 months (Fig. S1A). The SR cells showed larger IC_50_ values and higher mRNA and protein expression of multidrug resistance proteins compared with untreated cells, confirming their resistance to sorafenib (Fig. 1A; Fig. S1B, C). Additionally, both mRNA and protein levels of STIM1 were significantly up-regulated in Hep3B- and MHCC97H-SR cells compared with their parental (Con) cells (Fig. 1B, C). Considering that STIM1 is a crucial sensor for mediating Ca^2+^ influx in HCC cells,^17^ we also observed a significant enhancement in the Ca^2+^ influx signal in Hep3B- and MHCC97H-SR cells (Fig. 1D). Moreover, RNA next-generation sequencing was performed in SR- and Con-Hep3B cells to identify genes associated with SR (Fig. 1E). The differentially expressed genes were subjected to Kyoto Encyclopedia of Genes and Genomes (KEGG) enrichment analysis, which revealed a significant up-regulation of the “calcium signaling pathway” in SR-Hep3B (Fig. 1F). Furthermore, GSEA demonstrated a strong correlation between SR and calcium signaling pathway (Fig. 1G). Collectively, these findings suggest that enhanced STIM1-mediated SOCE may play a role in the acquired SR in HCC cell models.

**Figure 1:**
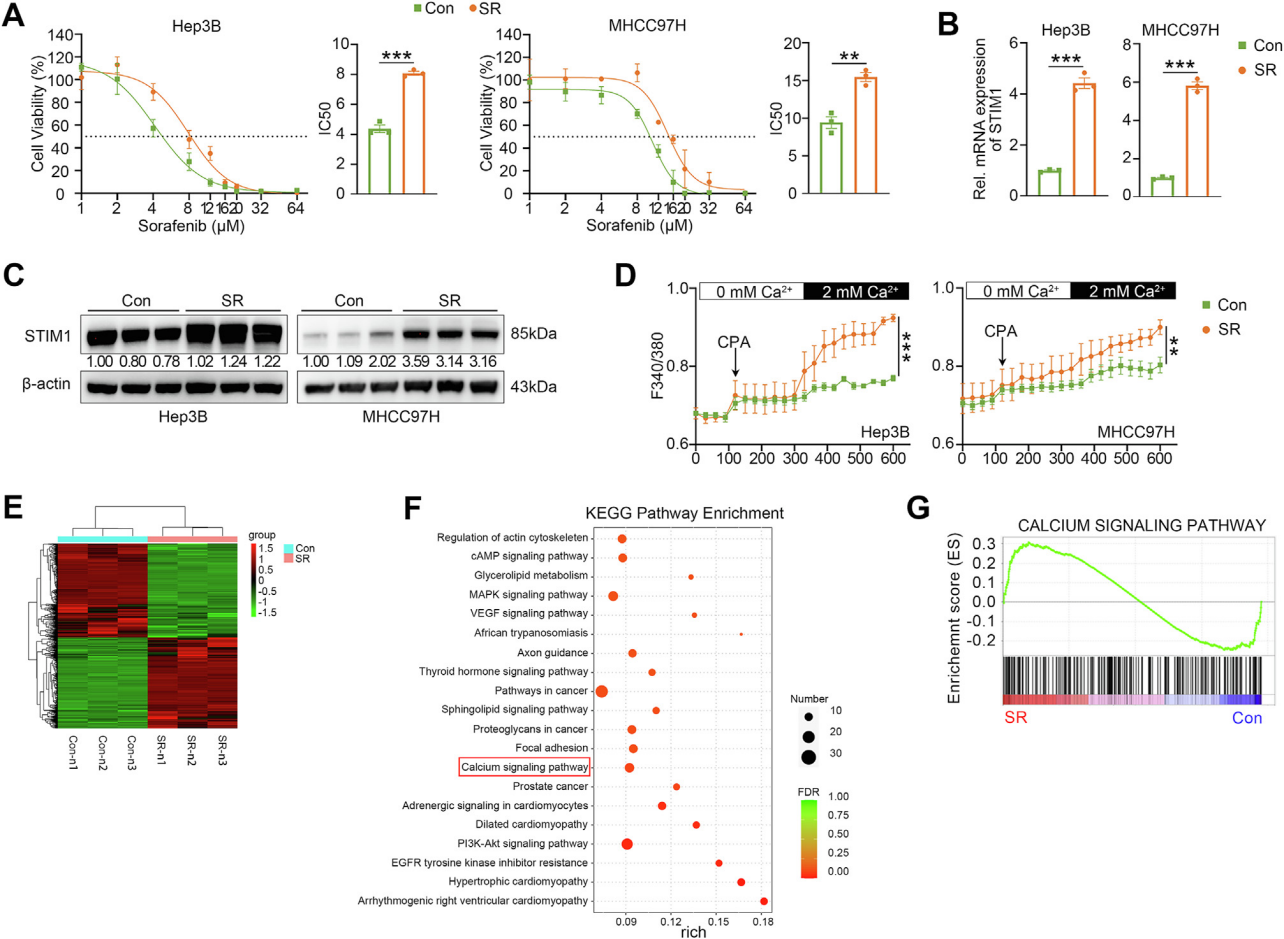
The level of STIM1 is elevated in SR-HCC cell models. **(A)** CCK-8 assays were used to examine the cell viability of sorafenib-parental (Con) and -resistant (SR) Hep3B and MHCC97H cells treated with various doses of sorafenib for 72 h. **(B)** Real-time quantitative PCR and **(C)** western blotting were applied to evaluate the mRNA and protein expression of STIM1 in Con- and SR-Hep3B and MHCC97H cells. β-actin was used as a loading control. **(D)** Calcium imaging was applied to analyze the calcium mobilization in Con- and SR-Hep3B and MHCC97H cells, respectively upon cyclopiazonic acid (CPA) (200 μM) stimulation. **(E)** The heatmap indicating the top 100 up-regulated and down-regulated genes between Con- and SR-Hep3B cells as detected in RNA next-generation sequencing with three biological duplicates. **(F)** Differentially expressed genes between Con- and SR-Hep3B cells were analyzed by Kyoto Encyclopedia of Genes and Genomes (KEGG) enrichment analysis. **(G)** GSEA demonstrated the correlation between SR and calcium signaling pathway in Hep3B SR cells. Data were expressed as mean ± standard error of the mean. ^∗∗^*P* < 0.01, ^∗∗∗^*P* < 0.001.

### STIM1 enhances SR in HCC cell lines

To investigate the role of up-regulated STIM1 in the development of acquired SR in HCC cells, we used CRISPR/Cas9 technology to knock down STIM1 in Hep3B- and MHCC97H-SR cells. The efficiency of knockdown was confirmed through western blotting analysis (Fig. 2A). The deletion of STIM1 resulted in impaired SOCE function in Hep3B- and MHCC97H-SR cells (Fig. 2B). Cell survival and cytotoxicity assays (Fig. 2C, D) showed that STIM1 knockdown significantly increased the sensitivity of Hep3B- and MHCC97H SR-cells to sorafenib. To further investigate the promoting effect of STIM1 on acquired SR in HCC cell lines, we conducted proliferation and sorafenib-induced cell death experiments using Con-, SR-, and SR STIM1 KD-Hep3B and MHCC97H cells. As depicted in Fig. S2A, the rate of proliferation was slower in STIM1 KD SR cells compared with SR HCC cells. However, when treated with sorafenib, the knockdown of STIM1 resulted in a more significant increase in cell death in SR HCC cells (Fig. 2E; Fig. S2B). This suggests that the heightened sensitivity to sorafenib induced by STIM1 knockdown primarily relies on sorafenib-induced cell death. Furthermore, *in vivo* experiments with the xenograft HCC model (Fig. 2F–I) demonstrated that STIM1 knockdown restored sensitivity to sorafenib in the SR-HCC cell models. These findings indicate that STIM1 deficiency effectively enhances the sensitivity of SR-HCC cells to sorafenib.

**Figure 2:**
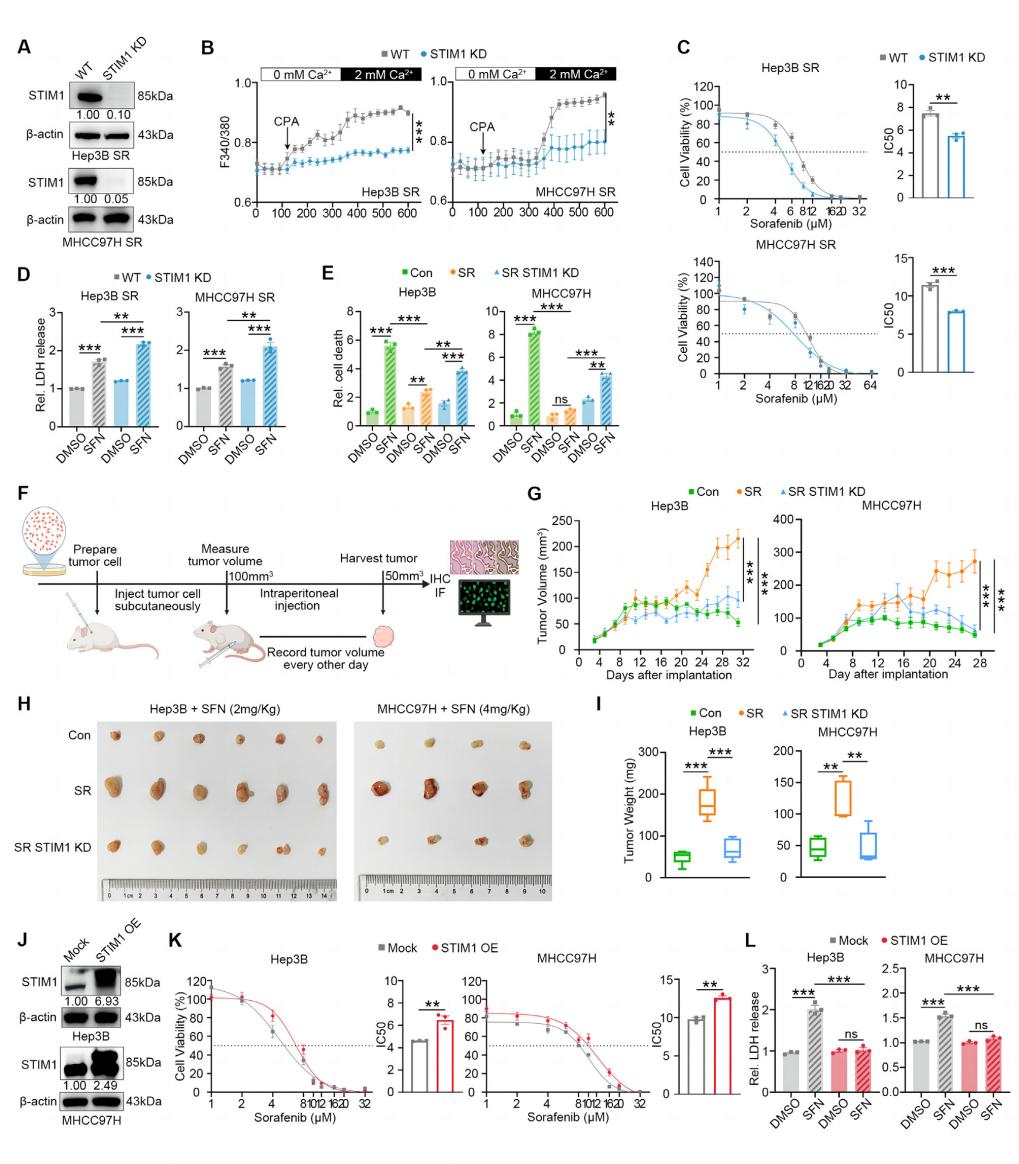
STIM1 regulates HCC resistance to sorafenib (SR). **(A)** Western blotting was applied to measure the protein level of STIM1 in the wildtype (WT)- and STIM1 knockdown (KD)-Hep3B and MHCC97H SR cells. β-actin was used as a loading control. **(B)** Calcium imaging was applied to analyze the calcium mobilization upon cyclopiazonic acid (CPA) (200 μM) stimulation in WT- and KD-Hep3B SR and MHCC97H SR cells, respectively. **(C)** The cell viability of WT- and KD-Hep3B SR and MHCC97H SR cells treated with various doses of sorafenib for 72 h were measured by CCK-8 assay. **(D)** The cell cytotoxicity of WT- and KD-Hep3B SR and MHCC97H SR cells treated with DMSO or sorafenib (SFN) (5 μM for group Hep3B SR, and 8 μM for group MHCC97H SR) for 72 h were determined by the release of lactate dehydrogenase (LDH). **(E)** The cell death levels of Con-, SR- and SR STIM1 KD-Hep3B and MHCC97H cells treated with DMSO or SFN (5 μM for group Hep3B, and 8 μM for group MHCC97H) for 72 h were assessed by flow cytometry following propidium iodide staining. **(F)** Schematic diagram (created by Biorender) of Hep3B and MHCC97H xenograft models. **(G**–**I)** B-NDG mice were injected subcutaneously with Con-, SR- and SR STIM1 KD-Hep3B and MHCC97H cells (1 × 10^7^ cells/mouse for the Hep3B group, and 5 × 10^6^ cells/mouse for the MHCC97H group). Then these mice were administrated with SFN (2 mg/kg/i.p. for the Hep3B group, 4 mg/kg/i.p. for the MHCC97H group, and once every other day). (G) The tumor volume of each mouse was calculated every two days and tumor growth curves were plotted. (H) Images of tumors harvested at the endpoint. (I) Tumor weight was measured at day 32 for the Hep3B group and 28 for the MHCC97H group. **(J)** Western blotting was applied to measure the protein level of STIM1 in the Mock- and STIM1 OE-Hep3B and MHCC97H cells. β-actin was used as a loading control. **(K)** The cell viability of Mock- and STIM1 OE-Hep3B and MHCC97H cells treated with the various doses of sorafenib for 72 h were measured by CCK-8 assay. **(L)** The cell cytotoxicity of Mock- and STIM1 OE-Hep3B and MHCC97H cells treated with DMSO or SFN (5 μM for Hep3B SR group, and 8 μM for MHCC97H SR group) for 72 h were determined by release of LDH. Data were expressed as mean ± standard error of the mean. ^∗∗^*P* < 0.01, ^∗∗∗^*P* < 0.001. ns represents no significant difference.

Besides, Hep3B and MHCC97H cells were infected with control (Mock) or STIM1 overexpression (OE) lentiviruses and validated by western blotting analysis (Fig. 2J). Conversely, the introduction of STIM1 led to an increase in the IC_50_ of parental HCC cells to sorafenib and a decrease in cell death (Fig. 2K, L). Collectively, the results from *in vitro* and *in vivo* experiments demonstrate that STIM1 promotes the development of acquired SR in HCC cells.

### STIM1 acts as a negative regulator of ferroptosis by raising GSH production

To investigate the contribution of STIM1 to HCC acquired SR, we performed transcriptome sequencing to detect the differentially expressed genes after the knockdown of STIM1 in SR-HCC cells (Fig. 3A). KEGG enrichment analysis revealed significant changes in GSH metabolism in STIM1 KD Hep3B SR cells (Fig. 3B). Furthermore, GSEA demonstrated a strong correlation between STIM1 and GSH metabolism (Fig. 3C). Treatment with sorafenib effectively reduced intracellular GSH content in Hep3B- and MHCC97H-Con cells. However, in their corresponding SR cells, GSH production did not significantly change after sorafenib treatment (Fig. S3A). Considering that GSH metabolism plays a crucial role in regulating ferroptosis^18^ and is consistent with previous studies,^19^ we also observed ferroptosis tolerance in our SR HCC cell models. Confocal microscopy and flow cytometry results showed no significant alteration in L-ROS levels in SR HCC cells treated with sorafenib compared with control cells (Fig. S3B, C). Similarly, MDA, a vital lipid peroxidation product, exhibited similar changes (Fig. S3D). Based on these findings, we hypothesized that STIM1 may attenuate ferroptosis by increasing GSH production, thereby inducing acquired SR in HCC.

**Figure 3:**
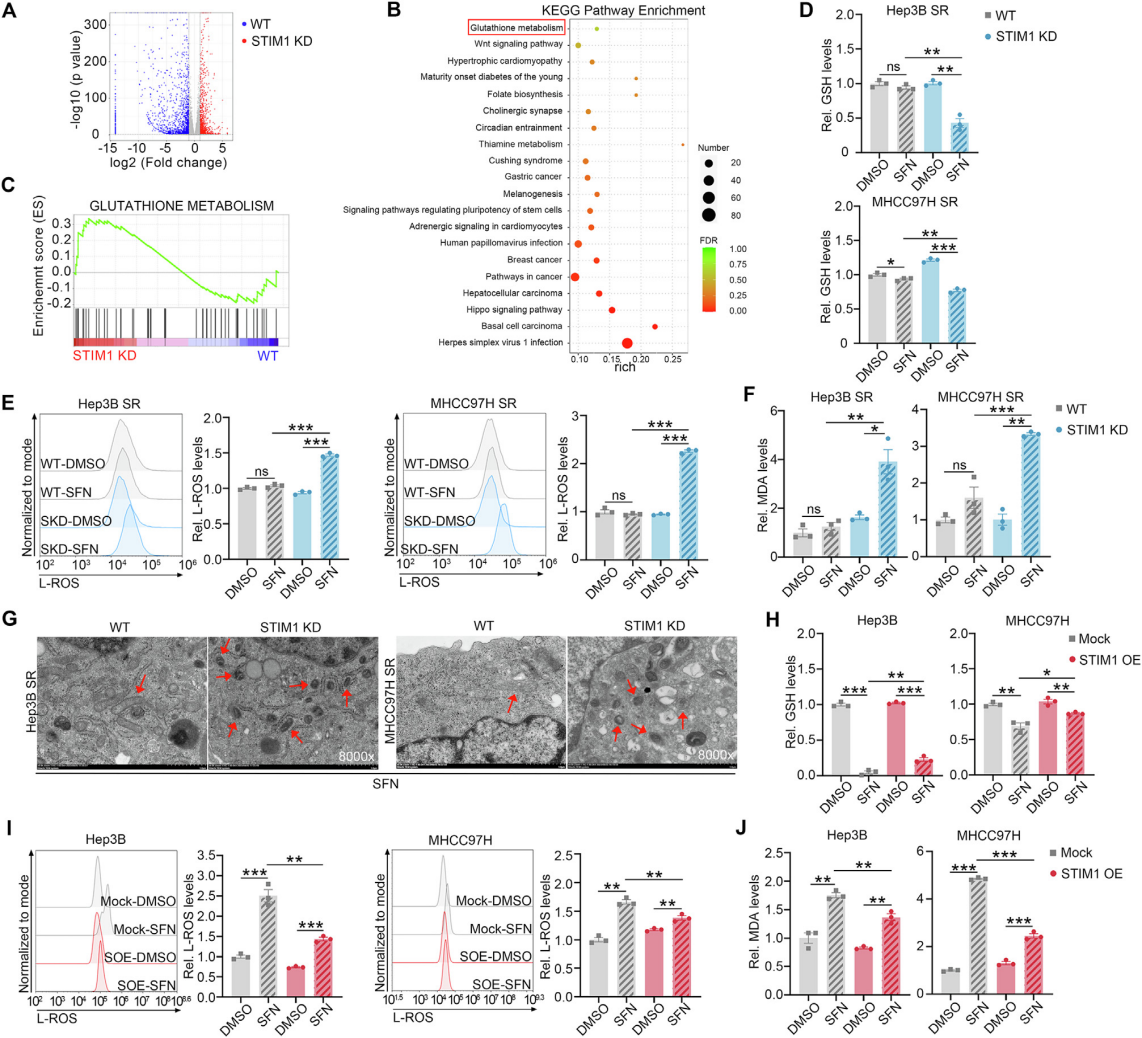
STIM1 blunts ferroptosis by elevating glutathione (GSH) levels. **(A)** The volcano plot showing differentially expressed genes between wild-type (WT)- and STIM1 KD-Hep3B SR cells based on absolute fold change >2 and *P* value < 0.05. **(B)** Differentially expressed genes between WT- and STIM1 KD-Hep3B SR cells were analyzed by KEGG enrichment analysis. **(C)** GSEA displaying correlations between STIM1 expression and glutathione metabolism in STIM1 KD Hep3B SR cells. **(D)** The WT- and STIM1 KD-Hep3B SR and MHCC97H SR cells were treated with DMSO or sorafenib (SFN) (5 μM for the Hep3B SR group, and 8 μM for the MHCC97H SR group) for 72 h, and the levels of glutathione (GSH) were assayed. **(E)** The levels of L-ROS in WT- and STIM1 KD-Hep3B SR and MHCC97H SR cells treated with DMSO or SFN (5 μM for Hep3B SR group, and 8 μM for MHCC97H SR group) for 72 h were assessed by flow cytometry following 665/676-BODIPY probe staining. **(F)** The levels of malondialdehyde (MDA) were quantified in WT- and STIM1 KD-Hep3B SR and MHCC97H SR cells treated with DMSO or SFN (5 μM for Hep3B SR group, and 8 μM for MHCC97H SR group) for 72 h. **(G)** Transmission electron microscopy was applied to observe the morphological feature of ferroptosis (mitochondrial size and mitochondrial membrane density) in WT- and STIM1 KD-Hep3B SR and MHCC97H SR cells treated with SFN (5 μM for Hep3B SR group, and 8 μM for MHCC97H SR group) for 72 h. **(H**–**J)** The GSH (H), L-ROS (C11-BODIPY probe staining) (I), and MDA (J) levels of Mock- and STIM1 OE-Hep3B and MHCC97H cells treated with DMSO or SFN (5 μM for Hep3B SR group, and 8 μM for MHCC97H SR group) for 72 h. Data were expressed as mean ± standard error of the mean. ^∗^*P* < 0.05, ^∗∗^*P* < 0.01, ^∗∗∗^*P* < 0.001. ns represents no significant difference.

Subsequently, a series of experiments were conducted to evaluate the impact of STIM1 on GSH production and ferroptosis. Upon knocking down STIM1 in Hep3B- and MHCC97H-SR cells, the suppressive effect of sorafenib on GSH production was noticeably restored (Fig. 3D). Furthermore, the deletion of STIM1 led to significantly increased levels of L-ROS and MDA after sorafenib treatment (Fig. 3E, F). Transmission electron microscopy analysis revealed distinct morphological changes associated with ferroptosis in STIM1 KD SR cells treated with sorafenib, including smaller mitochondria, reduced mitochondrial cristae, and wrinkled mitochondrial membranes (Fig. 3G). In contrast to Con-HCC cells, SR cells did not respond to the ferroptosis inducers Erastin and RSL-3. However, the deletion of STIM1 in SR cells restored their sensitivity to ferroptosis, indicating that STIM1 promotes ferroptosis tolerance in SR HCC cells (Fig. S3E). Considering the enhanced STIM1-mediated SOCE in SR HCC cells, we investigated whether inhibiting SOCE would enhance ferroptosis. In previous studies, SKF96365 has been confirmed as a strong inhibitor of STIM1-triggered SOCE.^20^ The staining of BODIPY C11 showed that when combined with sorafenib, SKF96365 significantly elevated L-ROS levels in SR HCC cells. However, this effect was reversed by ferrostatin-1, which is an inhibitor of ferroptosis (Fig. S3F, G). Conversely, the introduction of STIM1 in Hep3B and MHCC97H cells resulted in elevated GSH content, as well as reduced levels of L-ROS and MDA compared with the Mock group (Fig. 3H–J).

To investigate the role of STIM1 in SR in HCC cells, we examined its contribution to cell death pathways. Previous studies have shown that STIM1 can influence apoptosis and autophagy.^21^^,^^22^ In our experiments, we tested whether STIM1 inhibited ferroptosis, a form of programmed cell death, in SR HCC cells. We treated the cells with ferrostatin-1, a ferroptosis inhibitor, and observed a significant reduction in the sensitivity to sorafenib when STIM1 was deficient (Fig. S4A). However, other programmed cell death inhibitors, such as bafilomycin A1 (an autophagy inhibitor) and necrostatin-1 (a necroptosis inhibitor), were unable to rescue sorafenib-induced cell death in the absence of STIM1. Interestingly, the apoptosis inhibitor Z-VAD-FMK only slightly affected the sensitivity of STIM1-deleted cells to sorafenib. Moreover, flow cytometry analysis revealed that co-administration of SKF96365 did not significantly induce apoptosis in SR HCC cells (Fig. S4B). These results suggest that STIM1 reduces the accumulation of L-ROS by increasing GSH levels, thereby conferring resistance to ferroptosis in SR HCC cells.

### STIM1 enhances the transcription of SLC7A11

To investigate the molecular mechanisms by which STIM1 promotes GSH production, expression of ferroptosis-related genes was detected in both wild-type and STIM1 KD-Hep3B SR cells. Among these genes, SLC7A11,^23^ a cystine/glutamate reverse transporter protein, was the most significantly down-regulated gene in STIM1-deficient SR HCC cells. Moreover, deletion of STIM1 markedly decreased the expression of both mRNA and protein levels of SLC7A11 in Hep3B and MHCC97H SR-cells (Fig. 4A–C). Besides, the transplanted tumors of SR HCC cells showed higher STIM1 and SLC7A11 expression compared with the Con group. Meanwhile, STIM1 KD was followed by a dramatic reduction in SLC7A11 expression (Fig. 4D). Conversely, both mRNA and protein levels of SLC7A11 were greatly up-regulated after the lentiviral introduction of STIM1 in Hep3B and MHCC97H cells (Fig. 4E, F). More importantly, immunofluorescence assays illustrated higher SLC7A11 expression was relevant to higher STIM1 expression compared with tumor tissues from HCC patients with low STIM1 expression (Fig. 4G; Fig. S5A).

**Figure 4:**
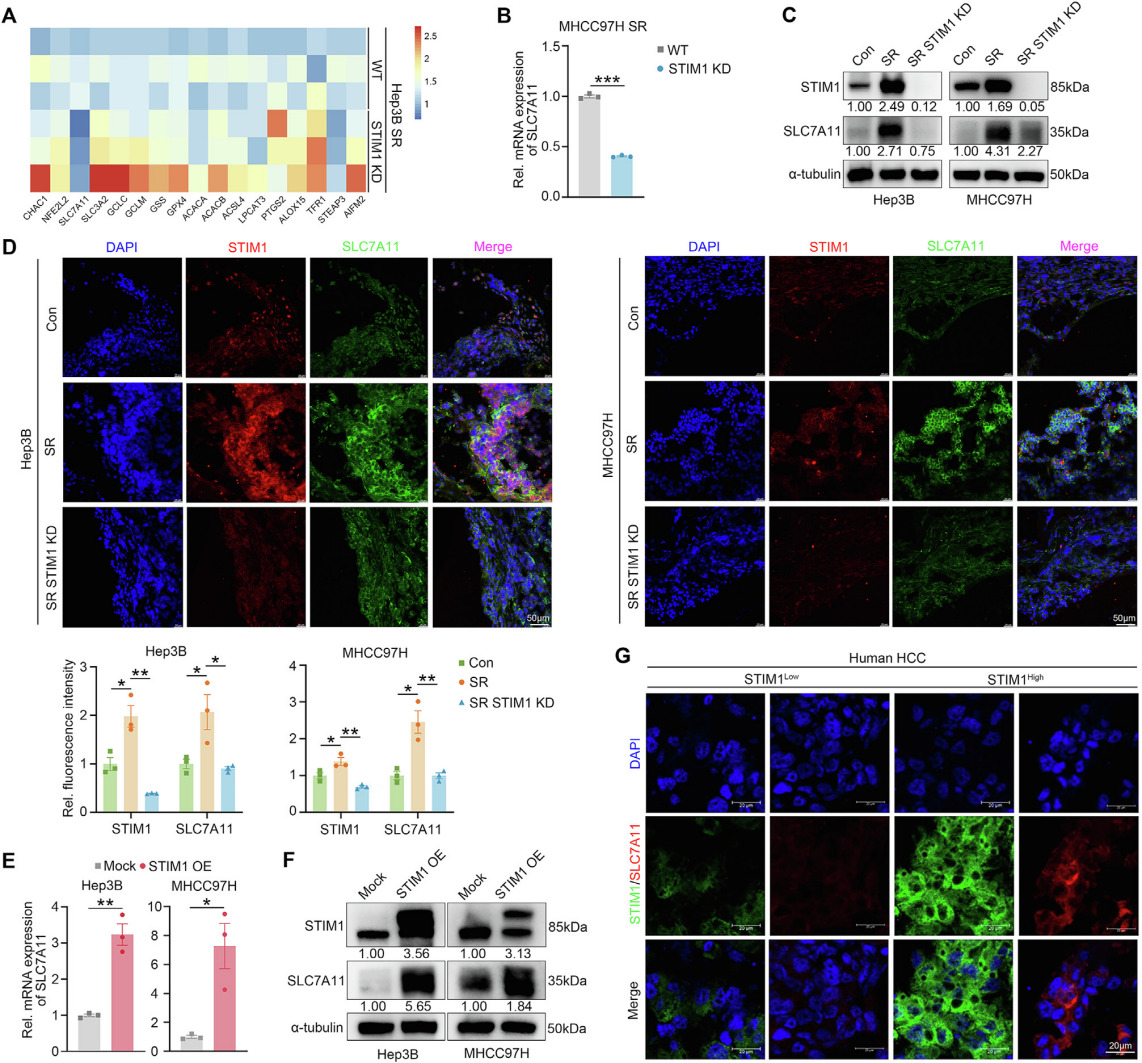
STIM1 promotes the expression of SLC7A11. **(A)** Heatmap of ferroptosis-related gene changes between wild-type (WT)- and STIM1 KD-Hep3B SR cells. **(B)** Real-time quantitative PCR and **(C)** western blotting were applied to evaluate the mRNA expression of SLC7A11 and protein expression of both STIM1 and SLC7A11 in WT- and STIM1 KD-SR cells. α-tubulin was used as a loading control. **(D)** The expression of STIM1 (red) and SLC7A11 (green) in the tumors of B-NDG mice were detected by immunofluorescence assays, and the cell nucleus was labeled with DAPI (blue). **(E)** The mRNA levels of *SLC7A11* and **(F)** protein levels of both STIM1 and SLC7A11 in WT- and STIM1 KD-Hep3B SR and MHCC97H SR cells were determined by real-time quantitative PCR and western blotting. α-tubulin was used as a loading control. **(G)** The expression of STIM1 (green) and SLC7A11 (red) in the tissues of patients were detected by immunofluorescence assays, and the cell nucleus was labeled with DAPI (blue). Data were expressed as mean ± standard error of the mean. ^∗^*P* < 0.05, ^∗∗^*P* < 0.01.

GPX4, a selenoprotein, acts as a ferroptosis inhibitor by relying on GSH to provide two electrons to neutralize L-ROS.^24^ The mRNA and protein levels of GPX4 in STIM1 KD Hep3B SR cells were not found to be significantly changed compared with the wild-type group (Fig. S5B, C). Furthermore, the genes involved in iron metabolism, such as SLC40A1, DMT1, TFR1, FTL, and STEAP3, which are important in regulating ferroptosis,^25^ were also not noticeably altered when STIM1 was lost (Fig. S5D). Additionally, FerroOrange probe staining revealed that there was no significant change in intracellular ferrous ions (Fe^2+^) levels in both STIM1 deficient SR cells and STIM1 overexpressing Hep3B cells (Fig. S5E). These findings suggest that STIM1 plays a role in facilitating SLC7A11 transcript expression in HCC.

### STIM1 promotes the transcriptional activation of SLC7A11 *via* SOCE-CaN-NFAT pathway

In order to investigate the relationship between STIM1 and SLC7A11, we conducted co-immunoprecipitation experiments in Hep3B- and MHCC97H-SR cells. Although the results showed that there was no protein interaction between STIM1 and SLC7A11 (Fig. 5A), our bioinformatics analysis using the JASPAR website revealed three potential NFAT-response elements (RE1 to 3) for NFAT within 1000 bp of the *SLC7A11* promoter (5′-TTTCC-3′) (Fig. 5B). NFAT is a nuclear transcription factor that has been previously reported to be involved in STIM1-mediated dephosphorylation through the activation of the SOCE-CaN (calcineurin) signaling pathway when the intracellular calcium pool is depleted.^26^ Once dephosphorylated, NFAT translocates to the nucleus and enhances the transcription of downstream genes, including *Cox-2*,^27^ *ENPP2*,^28^ and *Nanog*.^11^ Based on these findings, we hypothesized that STIM1 promotes SLC7A11 expression through the transcriptional activation of the SOCE-CaN-NFAT signaling pathway. To test this hypothesis, we compared the mRNA levels of SLC7A11 in STIM1 OE cells with Mock cells. Interestingly, the mRNA levels of SLC7A11 were significantly up-regulated in STIM1 OE cells, and this up-regulation could be inhibited by SKF96365, FK506 (a CaN inhibitor), or NFAT inhibitor-1 (an NFAT inhibitor) individually (Fig. 5C). Furthermore, our real-time quantitative PCR assay demonstrated that NFAT inhibitor-1 decreased the mRNA expression of SLC7A11 in Hep3B- and MHCC97H-SR cells (Fig. S6A). Furthermore, our flow cytometry results revealed that NFAT inhibitor-1 could increase the level of L-ROS in STIM1 OE HCC cells (Fig. S6B).

**Figure 5:**
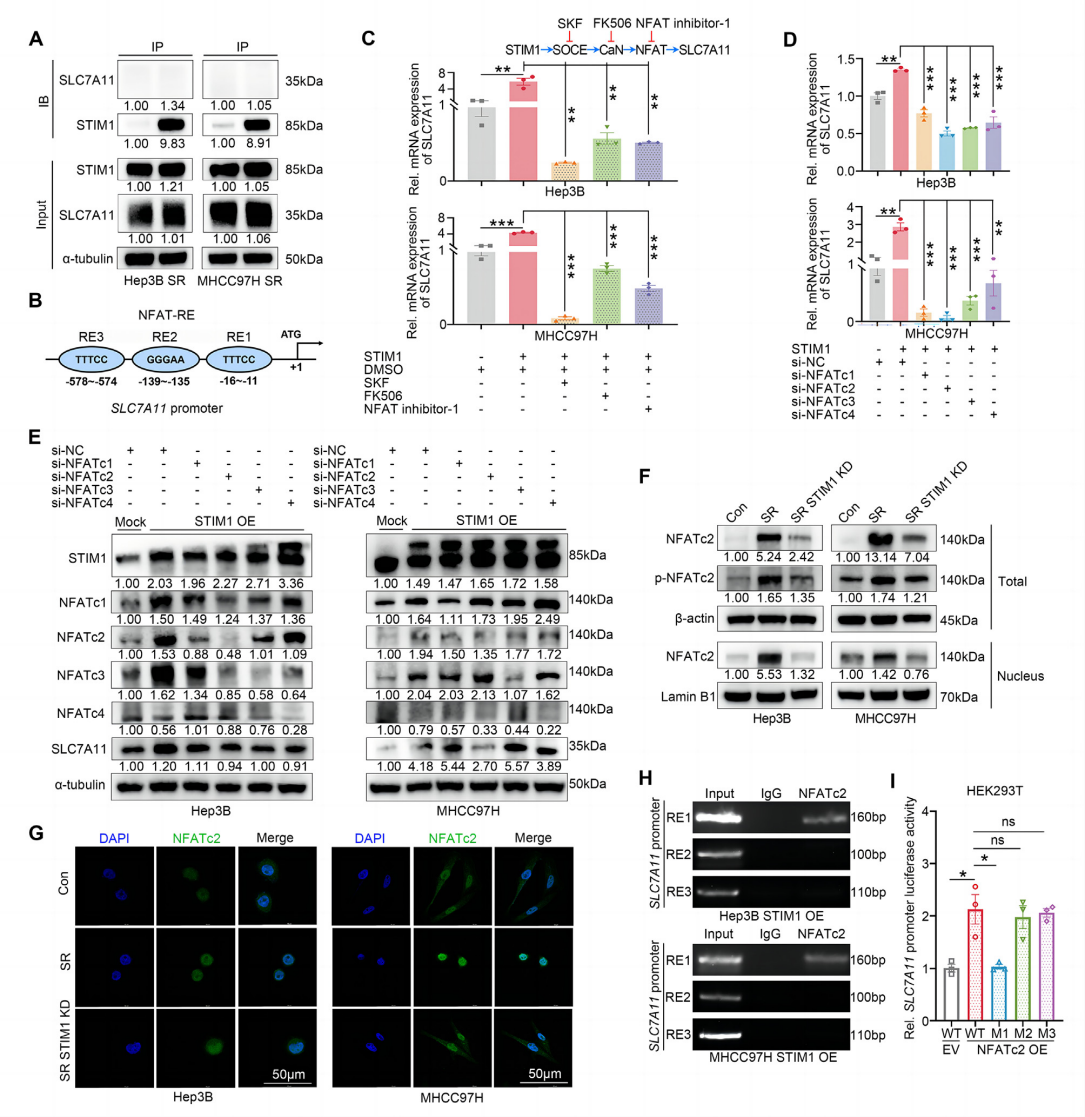
STIM1 transcriptionally activates *SLC7A11* expression by activating NFAT. **(A)** Co-immunoprecipitation assay of STIM1 and SLC7A11 in Hep3B SR and MHCC97H SR cells. α-tubulin was used as a loading control. **(B)** Bioinformatics analysis of the NFAT-response elements (REs) in the promoter of human *SLC7A11* through the JASPAR website. **(C)** Real-time quantitative PCR was applied to evaluate the SLC7A11 expression in Mock- and STIM1 OE-Hep3B and MHCC97H cells treated with DMSO or SKF96365 (SKF) (5 μM) or FK506 (100 nM) or NFAT inhibitor-1 (5 μM) for 24 h. **(D, E)** The mRNA levels of SLC7A11 (D) and protein levels of STIM1, NCATc1, NCATc2, NCATc3, NCATc4, and SLC7A11 (E) in Mock- and STIM1 OE-Hep3B and MHCC97H cells transfected with si-negative control (NC), si-NFATc1, si-NFATc2, si-NFATc3, or si-NFATc4 for 24 h were detected by real-time quantitative PCR and western blotting. α-tubulin was used as a loading control. **(F)** Western blotting was applied to quantify the protein levels of Total dephosphorylated and phosphorylated NFATc2 and nuclear NFATc2 in Con-, SR- and SR STIM1 KD-Hep3B and MHCC97H cells. **(G)** The expression of NFATc2 (green) in Con-, SR- and SR STIM1 KD-Hep3B and MHCC97H cells was detected by immunofluorescence assays, and the cell nucleus was labeled with DAPI (blue). **(H)** Chromatin immunoprecipitation assay of NFATc2 and *SLC7A11* promoter in STIM1 OE-Hep3B and MHCC97H cells, and IgG served as a negative control. **(I)** Luciferase reporter assay was applied to evaluate the activities of wild-type (WT) *SLC7A11* promoter and *SLC7A11* promoter containing single mutant NFAT (M1 to 3) in control (EV)- or NFATc2 OE-HEK-293T cells. Data were expressed as mean ± standard error of the mean. ^∗^*P* < 0.05, ^∗∗^*P* < 0.01, ^∗∗∗^*P* < 0.001. ns represents no significant difference.

To determine whether the isoforms of NFAT involved in STIM1 regulation of *SLC7A11* transcript levels and their molecular mechanism, we conducted a series of experiments. Firstly, siRNAs targeting specific isoforms of NFAT were transfected into STIM1 OE cells. The results demonstrated that silencing any of the NFAT isoforms significantly decreased *SLC7A1*1 mRNA levels (Fig. 5D). Notably, silencing NFATc2 had the strongest suppressive effect on SLC7A11 transcription. Additionally, the protein level of SLC7A11 showed the most significant down-regulation in STIM1 OE-Hep3B and MHCC97H cells when NFATc2 was silenced (Fig. 5E). Therefore, NFATc2 was chosen as a representative isoform for further investigation. Western blotting results demonstrated a reduction in the phosphorylation/dephosphorylation ratios of total NFATc2 protein in Hep3B (from 1.00 to 0.32) and MHCC97H (from 1.00 to 0.13) SR HCC cells, along with an increase in the amount of nuclear NFATc2 protein, indicating higher STIM1 expression. Conversely, knockdown of STIM1 led to an increase in the phosphorylation/dephosphorylation ratios of total NFATc2 protein in Hep3B SR (from 1.00 to 1.77) and MHCC97H SR (from 1.00 to 1.30) cells, accompanied by a decrease in nuclear NFATc2 protein levels (Fig. 5F). Additionally, compared with the control group, a majority of NFATc2 in SR HCC cells was localized in the nucleus, whereas deletion of STIM1 resulted in reduced nuclear localization of NFATc2 (Fig. 5G). These findings suggest that STIM1 plays a role in promoting the dephosphorylation and translocation of NFATc2.

To further explore the molecular mechanisms by which NFATc2 activates *SLC7A11* transcription, chromatin immunoprecipitation and luciferase reporter assays were performed. The chromatin immunoprecipitation results revealed a high enrichment of NFATc2 protein in the RE1 region of the *SLC7A1*1 promoter (Fig. 5H). Furthermore, luciferase reporter vectors containing wild-type or mutation-specific sites (M1 to 3) in the *SLC7A11* promoter region were constructed. The results showed that NFATc2 significantly increased the luciferase activity of the wild-type *SLC7A11* promoter, but failed to elevate the luciferase activity of the *SLC7A11* promoter with the M1 site mutation (Fig. 5I). Overall, these findings indicate that STIM1 transcriptionally activates *SLC7A11* through the activation of the SOCE-CaN-NFAT axis.

### SKF96365 enhances the anti-tumor activity of sorafenib in HCC with acquired SR *in vitro* and *in vivo*

**Figure 6:**
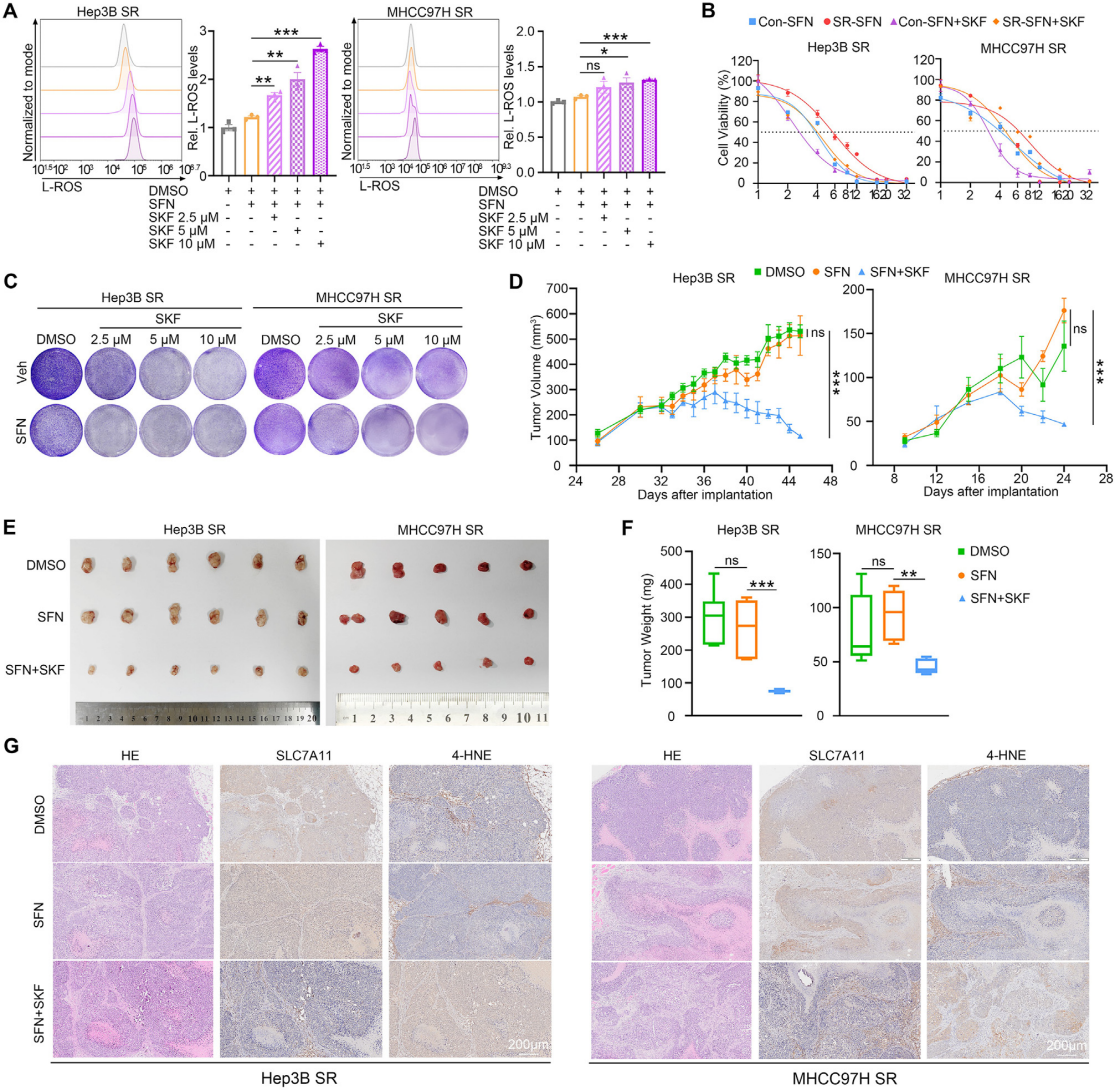
SKF96365 impairs ferroptosis tolerance and sorafenib resistance of SR HCC cells *in vitro* and *in vivo*. **(A)** The level of L-ROS in Hep3B- and MHCC97H-SR cells treated with DMSO or sorafenib (SFN) (5 μM for Hep3B SR group, and 8 μM for MHCC97H SR group) or SKF (2.5/5/10 μM) combined with SFN for 72 h were assessed by flow cytometry following C11-BODIPY probe staining. **(B)** The cell viability of Con- and SR-Hep3B and MHCC97H cells treated with various doses of SFN or SKF combined with SFN for 72 h. **(C)** Colony formation assays of Hep3B SR and MHCC97H SR cells treated with vehicle or SFN (5 μM for Hep3B SR group, and 8 μM for MHCC97H SR group) with increasing concentrations of SKF (2.5/5/10 μM). **(D**–**G)** B-NDG mice were injected subcutaneously with Hep3B SR and MHCC97H SR cells (1 × 10^7^ cells/mouse for Hep3B SR and 5 × 10^6^ cells/mouse for MHCC97H SR) and administrated with DMSO or SFN (2 mg/kg/i.p. for Hep3B group, and 4 mg/kg/i.p. for MHCC97H group, and once every other day) or SKF combined with SFN (10 mg/kg/i.p. and once every other day). (D) The tumor volume of each mouse was calculated every two days and tumor growth curves were plotted (*n* = 6). (E) Images of tumors harvested at the endpoint. (F) Tumor weight was measured at day 48 for Hep3B SR, and day 28 for MHCC97H SR. (G) Hematoxylin-eosin staining and immunohistochemistry staining of STIM1, NFATc2, SLC7A11, and 4-HNE in mice tumor. Data were expressed as mean ± standard error of the mean. ^∗^*P* < 0.05, ^∗∗^*P* < 0.01, ^∗∗∗^*P* < 0.001, ^∗∗∗∗^*P* < 0.0001. ns represents no significant difference.

The potential of SKF96365 to enhance the sensitivity of sorafenib in SR HCC cells was investigated in this study. Flow cytometry results demonstrated that co-administration of SKF96365 significantly increased L-ROS accumulation in a concentration-dependent manner (Fig. 6A). Moreover, cellular activity and colony formation assays indicated that combining SKF96365 with sorafenib significantly enhanced the cell-killing effect of sorafenib, also in a concentration-dependent manner, in both Hep3B- and MHCC97H-SR cells (Fig. 6B, C). In the SR-HCC xenograft model, the co-administration of SKF96365 with sorafenib effectively reduced xenograft tumor growth and weight compared with sorafenib alone (Fig. 6D–F). Hematoxylin-eosin staining and immunohistochemistry staining results revealed that the combination of SKF96365 with sorafenib down-regulated the expression of SLC7A11 and improved the level of 4-hydroxynonenal, an important indicator of lipid peroxidation, compared with sorafenib alone (Fig. 6G). Overall, these findings suggest that co-administration of SKF96365 could enhance the sensitivity of SR cells to sorafenib through ferroptosis.

## Discussion

**Figure 7:**
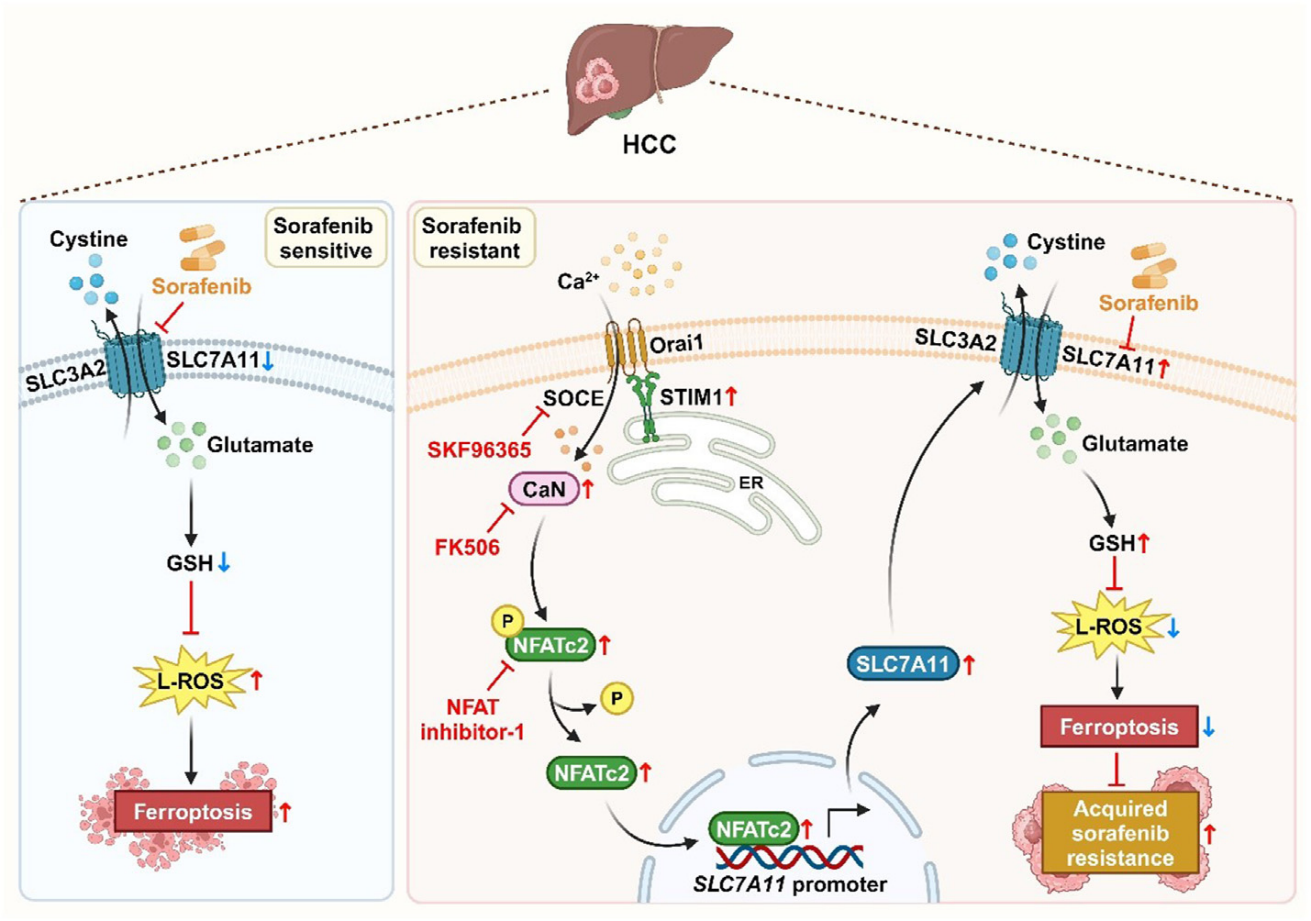
The schematic diagram illustrating the mechanism by which STIM1 promotes acquired sorafenib resistance in HCC. It is shown that STIM1 transcriptionally activates SLC7A11 through the up-regulation of the SOCE-CaN-NFAT signaling axis. As a result, the elevated expression of SLC7A11 enhances intracellular GSH production, which subsequently attenuates ferroptosis, ultimately leading to the development of acquired sorafenib resistance. CaN, calcineurin; GSH, glutathione; L-ROS, lipid reactive oxygen species; NFAT, nuclear factor of activated T cells; SLC7A11, solute carrier family 7 member 11; SLC3A2, solute carrier family 3 member 2; SOCE, store-operated calcium entry; STIM1, stromal interaction molecule 1.

Although sorafenib has been shown to prolong median overall survival in advanced HCC patients, only around 30 % of them can actually benefit from it. This is primarily due to the early development of acquired SR during systemic therapy.^29^ Therefore, improving the sensitivity of HCC cells to sorafenib has become a crucial clinical challenge. Various mechanisms have been identified that contribute to the development of acquired resistance in HCC. These include epigenetic modifications, such as non-coding RNAs and methylation modifications, which make HCC less susceptible to sorafenib.^30^ Additionally, ABC transporters and exosomes can prevent sorafenib from effectively binding to HCC.^31^ Besides, the properties and components of the tumor microenvironment, such as hypoxia, tumor-associated macrophages, and CD8^+^ T cells, can also reduce HCC sensitivity to sorafenib.^32^^,^^33^ Furthermore, regulatory cell death processes like autophagy and ferroptosis have been found to promote acquired SR in HCC.^34^^,^^35^ Despite these challenges, resolving SR in HCC patients remains a key objective. In this study, we investigated the role of STIM1-mediated SOCE in SR. We observed that STIM1 expression and SOCE function were significantly enhanced in SR HCC cells. Moreover, we found that STIM1 induced SR by suppressing ferroptosis. Specifically, STIM1 accelerated the transcription of *SLC7A1*1 by activating the STIM1-SOCE-CaN-NFAT axis. The up-regulated SLC7A11 then blunted ferroptosis by increasing GSH production, thus leading to SR. Importantly, we demonstrated that targeting this axis using the SOCE inhibitor SKF96365 greatly enhanced the sensitivity of sorafenib, providing a potential molecular basis for overcoming SR in HCC patients (see Fig. 7 for a visual summary).

It is widely believed that an excess of Ca^2+^ can trigger apoptosis in various types of tumor cells and immune cells, including pancreatic cancer and macrophages.^36^^,^^19^ In our SR-HCC cell models, the co-administration of SKF96365 did not significantly affect apoptosis but instead strongly induced ferroptosis. Furthermore, the inhibitory effect of the ferroptosis inhibitor ferrostatin-1 on sorafenib-induced cell death in STIM1-deficient SR cells was much greater than that of the apoptosis inhibitor Z-VAD-FMK. These findings suggest that in our constructed SR HCC cell models, the STIM1-mediated SOCE-induced acquired SR through ferroptosis rather than apoptosis, highlighting the importance of ferroptosis as a crucial mechanism in SR.

The important function of SLC7A11 is to import cystine for GSH biosynthesis in order to neutralize L-ROS and inhibit ferroptosis. This study demonstrates that SLC7A11 can be activated through transcriptional regulation or protein interactions. For instance, Yin Yang 1 (YY1) and YY2 competitively bind to the same DNA binding site in the *SLC7A11* promoter. YY1 promotes the transcription of *SLC7A11*, while YY2 represses it. The PI3K/AKT/NRF2 axis, when stimulated, activates the transcription of ATP-binding cassette transporter C family (ABCC) 5, resulting in increased expression of ABCC5 proteins. These proteins interact with the SLC7A11 protein in the cell membrane, enhancing the function of SLC7A11. In this work, we identified that the up-regulation of STIM1 led to the transcriptional activation of *SLC7A11* through the SOCE-CaN-NFAT signaling pathway. Overall, our study elucidates the regulatory relationship between STIM1-mediated Ca^2+^ signaling and SLC7A11.

Numerous studies have demonstrated the strong connection between ferroptosis and various cancer treatments, including chemotherapy, radiotherapy, targeted therapy, and immunotherapy. Targeting ferroptosis shows great promise as an effective anti-tumor therapy.^37^ For example, the chemotherapeutic agent altretamine can induce ferroptosis by attenuating GPX4.^38^ Similarly, the targeted agent sorafenib inhibits SLC7A11 to promote ferroptosis.^39^ Immunosuppressive cells like M2 macrophages, Treg cells, and myeloid-derived suppressor cells suppress ferroptosis through high expression of GPX4.^40^ Moreover, combining GPX4 inhibitors with anti-PD1 has been found to effectively induce ferroptosis in triple-negative breast cancer, demonstrating superior therapeutic efficacy compared with monotherapy.^41^ In our study, we discovered that STIM1 restrains ferroptosis by enhancing SLC7A11 expression through the SOCE-CaN-NFAT axis. This finding highlights the potential of targeting this pathway to improve ferroptosis in anti-tumor therapies.

The STIM1-SOCE-NFAT axis was identified as a significant factor in ferroptosis tolerance in our study. To investigate this pathway, we utilized SKF96365 as a representative inhibitor. Our findings showed that blocking this axis effectively restored sensitivity to sorafenib in SR HCC cells. Additionally, it is important to note that Ca^2+^ signaling serves as a fundamental second messenger involved in various physiological activities within the body. STIM1-mediated SOCE not only impacts tumor cells but also plays a role in the tumor microenvironment. For instance, STIM1 promotes the activation, expansion, and differentiation of CD8^+^ T cells,^42^ enhances the secretion of perforin, granzyme, and tumor necrosis factors by neutrophils,^43^ and improves the antigen-presenting effects of dendritic cells.^44^ However, it is worth considering the effects on the immune system when continuing research with SKF96365 since we conducted *in vivo* experiments using immunodeficient mice. Therefore, it is crucial to consider potential side effects on normal cells and the tumor microenvironment when utilizing SKF96365.

In conclusion, our findings demonstrate that up-regulated STIM1 activates SLC7A11 transcription through the SOCE-CaN-NFAT axis, thereby reducing ferroptosis and contributing to SR in HCC cells. The use of the SOCE promotes ferroptosis and alleviates SR, making it a potential strategy to overcome SR in HCC patients.

## Ethics declaration

The use of clinical specimens in this study was approved by the Ethics Committee of Chongqing University Cancer Hospital (Chongqing, China), and informed written consent was obtained from all participants (Ethics Approval Number: CZLS2023239-A). All experimental procedures were approved by the Animal Ethics Committee of Chongqing University Cancer Hospital and conducted in accordance with the guidelines of the National Institutes of Health regarding animal welfare.

## Author contributions

Ran Ren: data curation, validation, visualization, formal analysis, and writing–original draft. Yu Chen: data curation. Yu Zhou: resources and methodology. Juan Lei: methodology. Jingchun Wang: methodology. Luyao Shen, Yang Chen, Xudong Liu, Nan Zhang, and Dongqin Zhou: Investigation. Huakan Zhao: writing–review & editing and funding acquisition. Yongsheng Li: project administration, conceptualization, supervision, writing–review & editing, and funding acquisition. All authors reviewed the manuscript, provided feedback, and approved the manuscript in its final form.

## Conflict of interests

The authors declared that they had no known competing financial interests or personal relationships that could have appeared to influence the work reported in this paper.

## Funding

This work was supported by the Major International (Regional) Joint Research Program of the National Natural Science Foundation of China (No. 81920108027), the National Natural Science Foundation of China (No. 82273212), Chongqing Postgraduate Research and Innovation Project (China) (No. CYS23130), Chongqing Outstanding Youth Science Foundation (China) (No. cstc2020jcyj-jqX0030), and Chongqing Science and Technology Innovation Leading Talent Support Program (China) (No. cstc2021ycjh-bgzxm0073).

## Data availability

To obtain the RNA sequencing data, check the website with the accession numbers GSE248769 and GSE248770 on https://www.ncbi.nlm.nih.gov/bioproject. Other data that support the findings of this study are available from the corresponding author upon reasonable request.

## Figures and Tables

**figs1:**
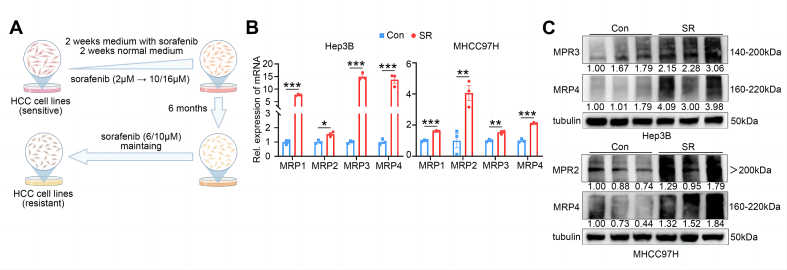


**figs2:**
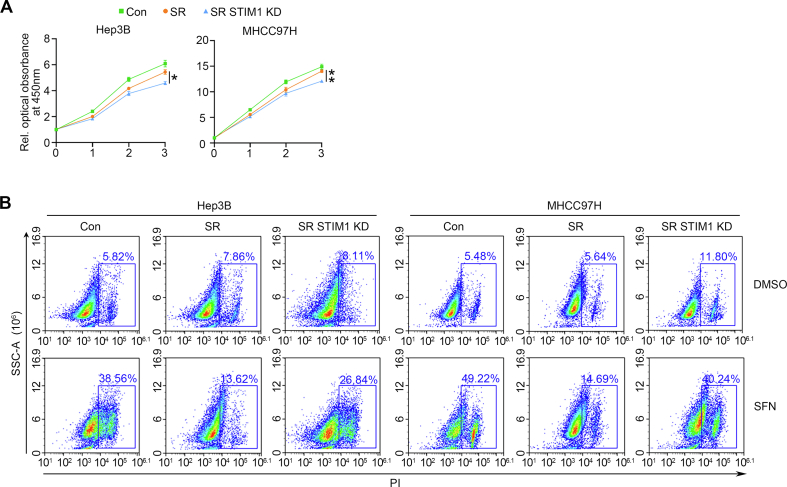


**figs3:**
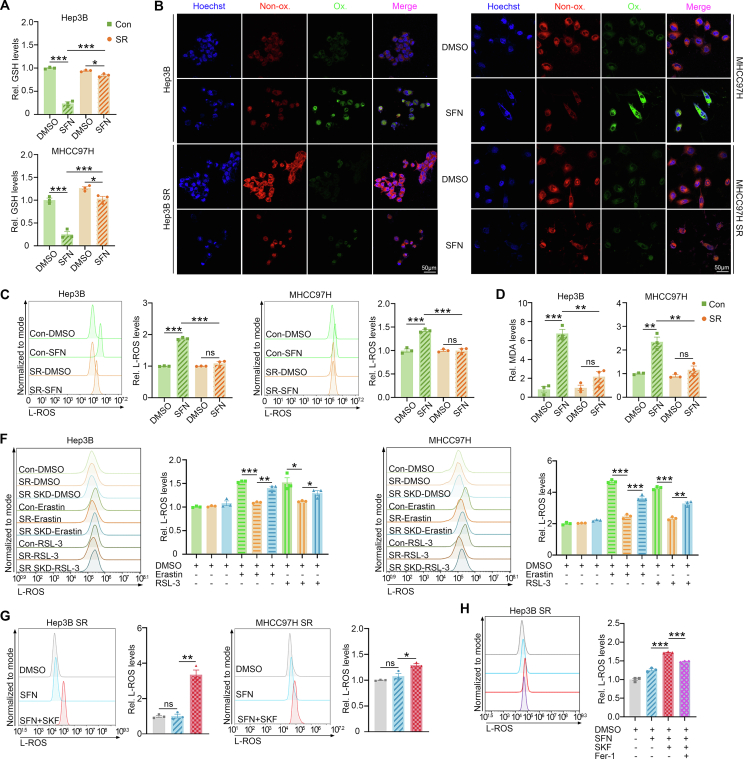


**figs4:**
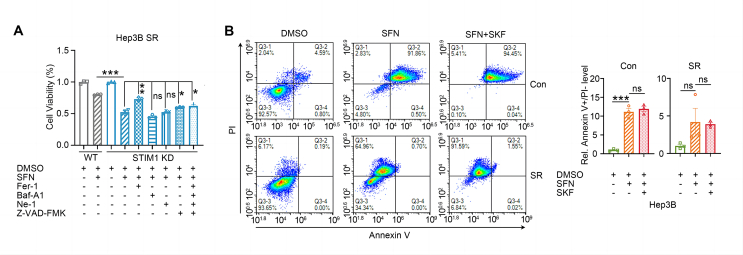


**figs5:**
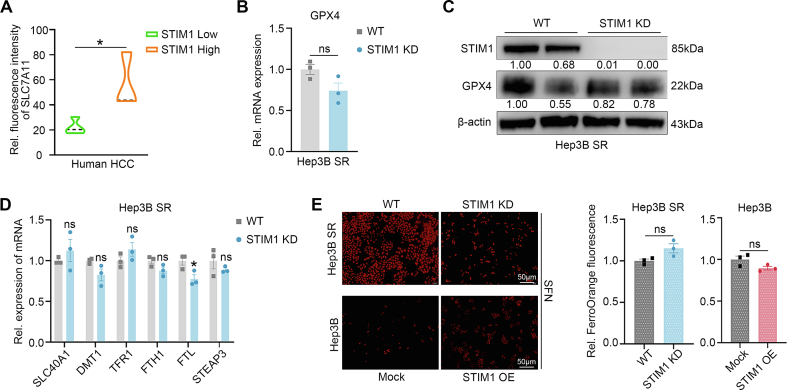


**figs6:**