# Supplementary material for: STIM1 promotes acquired resistance to sorafenib by attenuating ferroptosis in hepatocellular carcinoma
Source: Genes Dis. 2024 Mar 28;11(6):101281. doi: 10.1016/j.gendis.2024.101281 (PMC11402164; doi:10.1016/j.gendis.2024.101281)
Supplement: Multimedia component 1 [file mmc1.docx]

# Supplementary Materials

**Supplementary Figures**


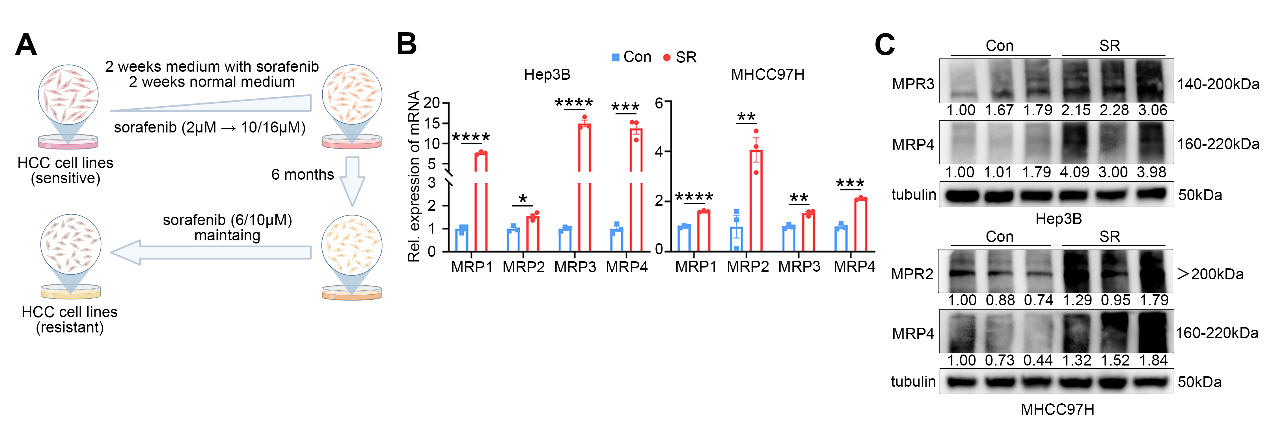


**Supplementary Figure 1.** Establishment and validation of acquired resistance to sorafenib in HCC cell lines. (A) The establishment of acquired resistance to sorafenib in Hep3B and MHCC97H cells (created by Biorender). (B) The mRNA expression of MRP1, MRP2, MRP3 and MRP4 in sorafenib -parental (Con) and -resistant (SR) Hep3B and MHCC97H cells were examined by RT-qPCR. (C) WB analysis evaluated the protein expression of MRP3/4 in Con- and SR- Hep3B cells and MRP2/4 in Con- and SR- MHCC97H cells. Data are expressed as means ± SEM. **p < 0.01, ***p < 0.001.


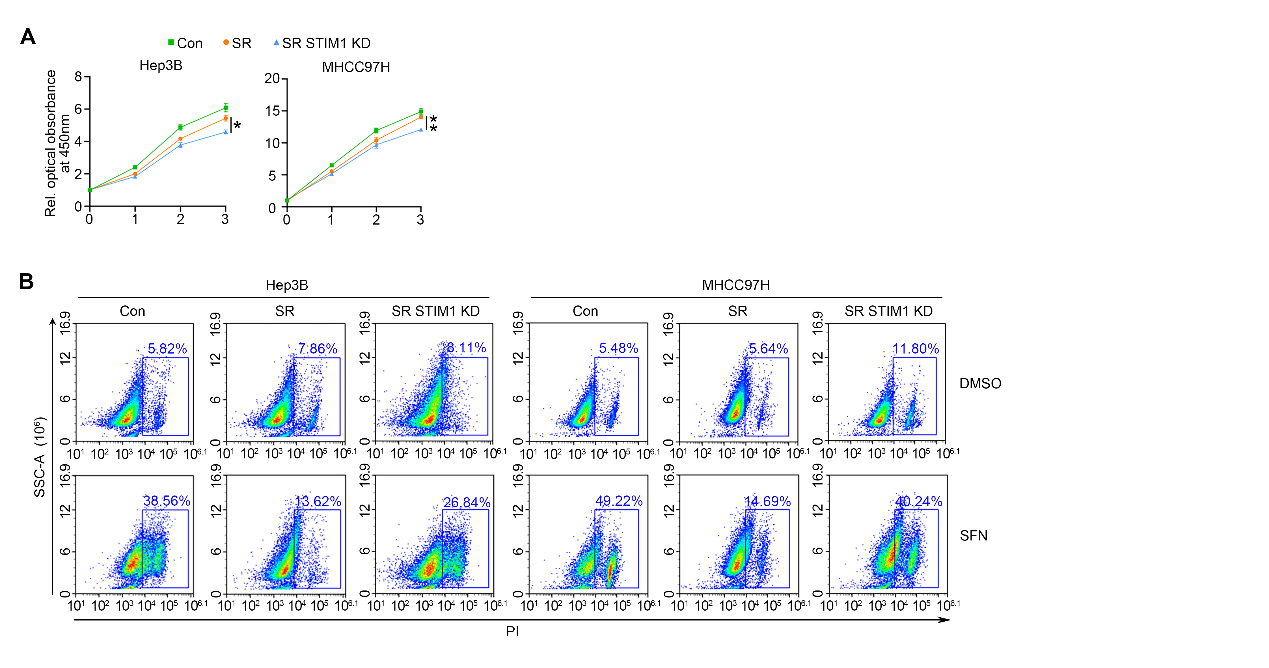


**Supplementary Figure 2.** The proliferation and sorafenib-induced cell death levels in Con-, SR- and SR STIM1 KD- Hep3B and MHCC97H cells. (A) The proliferation rate of Con-, SR- and SR STIM1 KD- Hep3B and MHCC97H cells were examined by CCK-8 assay. (B) Flow cytometry with Propidium Iodide (PI) staining was applied to measure the sorafenib-induced cell death levels in Con-, SR- and SR STIM1 KD- Hep3B and MHCC97H cells.


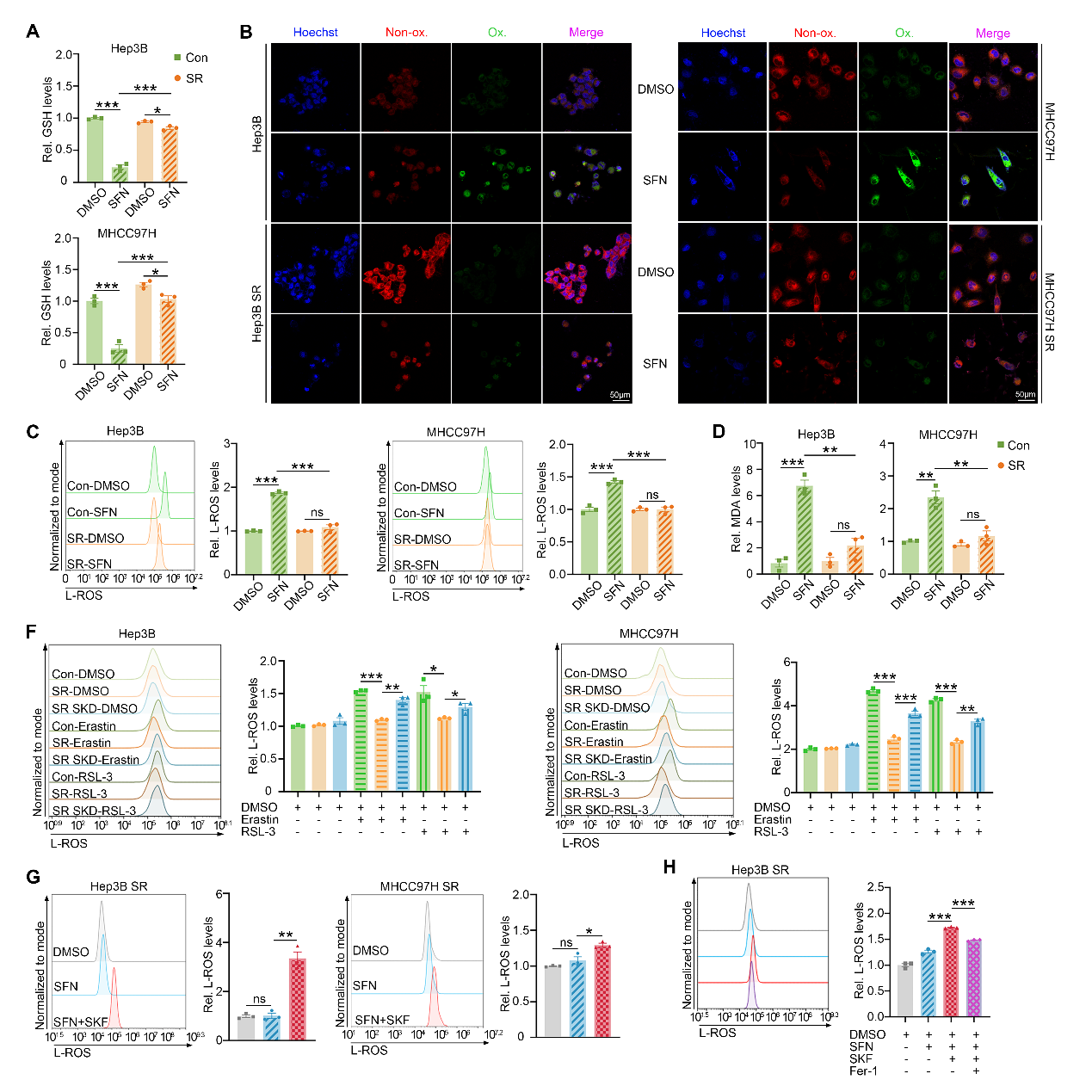


**Supplementary Figure 3.** The levels of ferroptosis in Con- and SR- Hep3B and MHCC97H cells, and L-ROS levels in Hep3B- and MHCC97H- SR cells when combined with SKF96365. (A) The Con- and SR- Hep3B SR and MHCC97H SR cells were treated with DMSO or sorafenib (SFN), and the levels of glutathione (GSH) were assayed. Confocal microscopy (B) and flow cytometry (C) estimated the alterations of L-ROS in Con- and SR- Hep3B SR and MHCC97H SR cells following DMSO or SFN treating. (D) The levels of malondialdehyde (MDA) were quantified in Con- and SR- Hep3B and MHCC97H cells treated with DMSO or SFN. (E) The levels of L-ROS in Con-, SR- and SR STIM1 KD- Hep3B and MHCC97H cells treated with Erastin (40μM) or RSL-3 (4μM) were assessed by flow cytometry. (F) The levels of L-ROS in Hep3B- and MHCC97H- SR cells treated with DMSO or SFN or SKF96365 (SKF) combined with SFN were assessed by flow cytometry. (G) The levels of L-ROS in Hep3B SR treated with DMSO or SFN or SFN combined with SKF or SFN combined with SKF and Fer-1 were assessed by flow cytometry. Data are expressed as means ± SEM. *p < 0.05, **p < 0.01, ***p < 0.001. ns represents no significant difference.


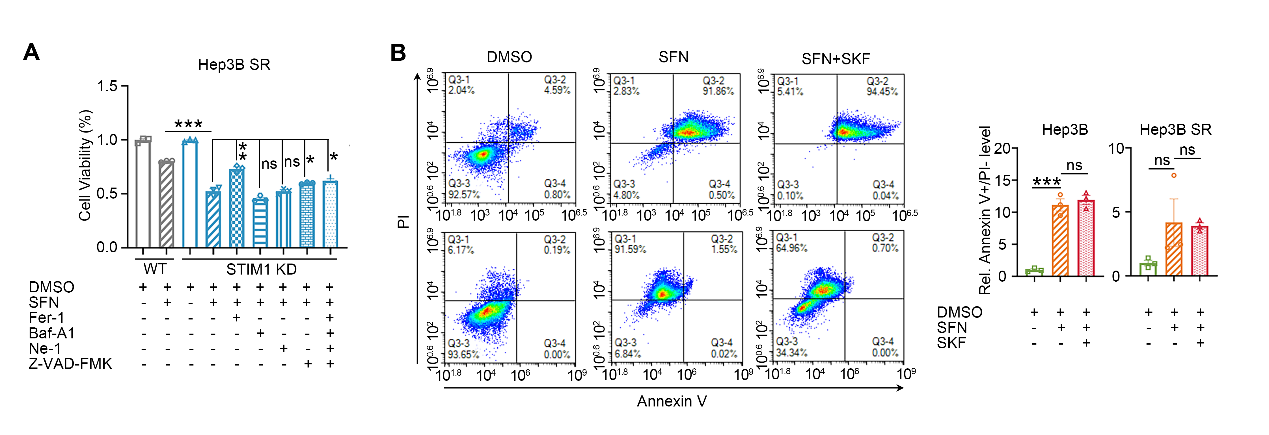


**Supplementary Figure 4.** The level of apoptosis in Hep3B SR cells did not alter significantly comparing with Hep3B cells. (A) The cell viability of WT- and STIM1 KD- Hep3B SR cells treated with DMSO or SFN or SFN combined with ferrostatin-1 (Fer-1) or bafilomycin A1 (Baf-A1) or necrostatin-1 (Ne-1) or Z-VAD-FMK were measured by CCK-8 assay. (B) Flow cytometry was applied to measure the apoptosis levels in Con- and SR- Hep3B cells treated with DMSO or SFN or SFN combined with SKF with the simultaneous staining of Annexin V and Propidium Iodide (PI). Data are expressed as means ± SEM. *p < 0.05, **p < 0.01, ***p < 0.001. ns represents no significant difference.


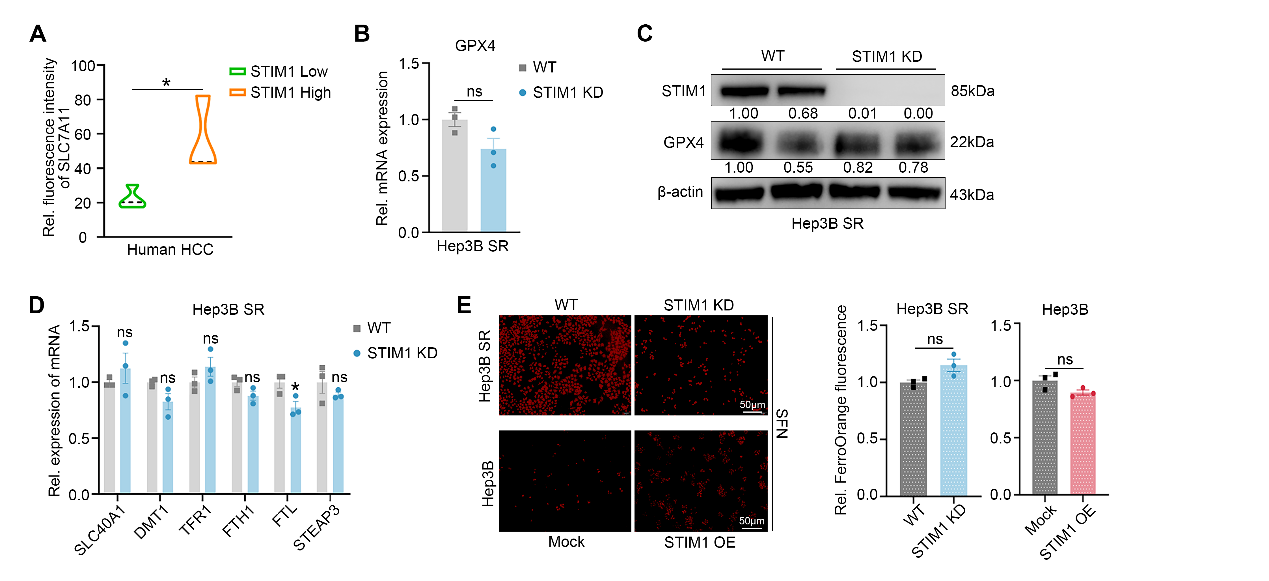


**Supplementary Figure 5.** STIM1 promotes SLC7A11 expression. (A) The mean fluorescence intensity of SLC7A11 in samples from 7 HCC patients. (B) The mRNA levels of GPX4 and (C) protein levels of both STIM1 and GPX4 in WT- and STIM1 KD- Hep3B SR cells were detected by RT-qPCR and WB. β-actin was used as a loading control. (D) RT-qPCR was applied to measure the mRNA levels of SLC40A1, DMT1, TFR1, FTH1, FTL and STEAP3 in WT- and STIM1 KD- Hep3B SR cells. (E) The level of intracellular ferrous ions (Fe^2+^) in WT- and STIM1 KD- Hep3B SR cells and Mock- and STIM1 OE- Hep3B cells were measured by FerroOrange probes. Data are expressed as means ± SEM. *p < 0.05. ns represents no significant difference.


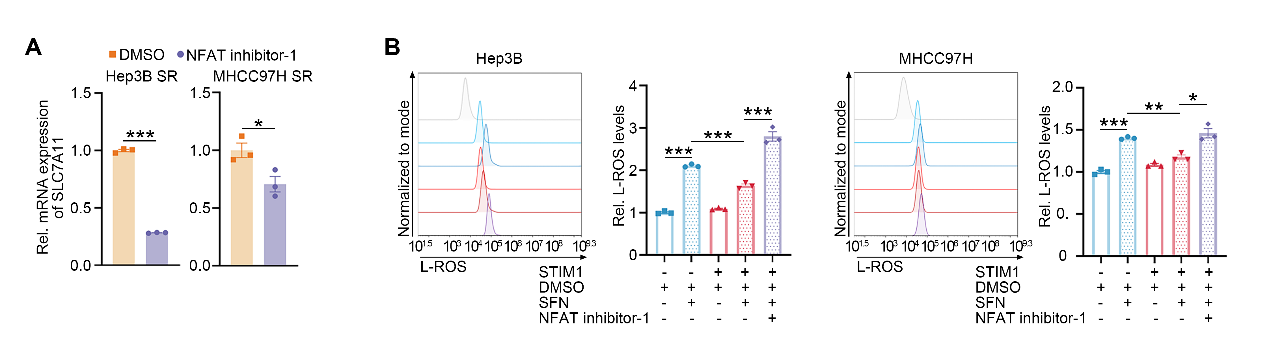


**Supplementary Figure 6.** NFAT inhibitor decreased SLC7A11 levels in SR HCC cells, and elevated L-ROS levels in STIM1 OE HCC cells. (A) The mRNA levels of SLC7A11 in Hep3B SR and MHCC97H SR cells treated with DMSO or NFAT inhibitor-1 were measured by RT-qPCR. (B) The level of L-ROS in Mock- and STIM1 OE- Hep3B and MHCC97H cells treated with DMSO or SFN (5 μM for group Hep3B, and 8 μM for group MHCC97H) or NFAT inhibitor-1 (5 μM) combined with SFN for 48 h were assessed by flow cytometry following C11-BODIPY probe staining. Data are expressed as means ± SEM. *p < 0.05, **p < 0.01, ***p < 0.001.

**Supplementary Tables**

**Supplementary Table 1.** Human sample information.

| **No.** | **Age**  **(year-old)** | **Sex** | **Tumor Size (cm*cm)** | **Procedure** | **BCLC Stage** | **TNM Stage** | **HBsAg** | **Tumor Type** |
| --- | --- | --- | --- | --- | --- | --- | --- | --- |
| # 1 | 45 | Male | 11.1*9.0 | Surgical  resection | I B | cT1bN0M0 | + | HCC |
| # 2 | 60 | Male | 4.4*5.0 |  | IV A | cT1bN1M0 | + |  |
| # 3 | 59 | Male | 2.8*2.5 |  | I B | cT1bN0M0 | + |  |
| # 4 | 48 | Male | 3.2*2.7 |  | II A | cT2N0M0 | + |  |
| # 5 | 58 | Male | 2.8*1.4 |  | I A | cT1aN0M0 | + |  |
| # 6 | 57 | Male | 4.0*3.3 |  | I B | cT1bN0M0 | + |  |
| # 7 | 58 | Male | 3.8*3.8 |  | II A | cT2N0M0 | + |  |

BCLC: Barcelona Clinic Liver Cancer

**Supplementary Table 2.** Primers for RT-qPCR.

| **Name (human)** | **Sequence of forward primer** | **Sequence of reverse primer** |
| --- | --- | --- |
| MRP1 | AAGGAGGTACTAGGTGGGCTT | CCAGTAGGACCCTTCGAGC |
| MRP2 | CCCTGCTGTTCGATATACCAATC | TCGAGAGAATCCAGAATAGGGAC |
| MRP3 | TCGAGAGAATCCAGAATAGGGAC | GCAAGACCATGAAAGCGACTC |
| MRP4 | AGCTGAGAATGACGCACAGAA | ATATGGGCTGGATTACTTTGGC |
| STIM1 | AGTCACAGTGAGAAGGCGAC | CAATTCGGCAAAACTCTGCTG |
| SLC7A11 | GCGTGGGCATGTCTCTGAC | GCTGGTAATGGACCAAAGACTTC |
| SLC3A2 | CTGGTGCCGTGGTCATAATC | GCTCAGGTAATCGAGACGCC |
| CHAC1 | CTGTGGATTTTCGGGTACGG | CTGTGGATTTTCGGGTACGG |
| NFE2L2 | CTGTGGATTTTCGGGTACGG | AGGCATCTTGTTTGGGAATGTG |
| GCLC | CTACCACGCAGTCAAGGACC | CTACCACGCAGTCAAGGACC |
| GCLM | AGGAGCTTCGGGACTGTATCC | AGGAGCTTCGGGACTGTATCC |
| GPX4 | GAGGCAAGACCGAAGTAAACTAC | CCGAACTGGTTACACGGGAA |
| GSS | GGAACATCCATGTGATCCGAC | GCCATCCCGGAAGTAAACCA |
| ACACA | TCACACCTGAAGACCTTAAAGCC | AGCCCACACTGCTTGTACTG |
| ACACB | CGCTCACCAACAGTAAGGTGG | GCTTGGCAGGGAGTTCCTC |
| FSP1 | ACAACTTCCGGCAGGGGC | GCTGTTGATCTTGATGCCGG |
| ACSL4 | ACTGGCCGACCTAAGGGAG | GCCAAAGGCAAGTAGCCAATA |
| LPCAT3 | GGAGACCTACCTCATCCACCT | CGGCCCATTAGTCGAAGGA |
| ALOX15 | GGGCAAGGAGACAGAACTCAA | CAGCGGTAACAAGGGAACCT |
| AIFM2 | AGAACCGGATGGTGTTGCTAC | CACCTCGTTAAACTTGCCAGG |
| PTGS2 | ATGCTGACTATGGCTACAAAAGC | TCGGGCAATCATCAGGCAC |
| TFR1 | ACTTGCCCAGATGTTCTCAG | GTATCCCTCTAGCCATTCAGTG |
| STEAP3 | GGCTGCTCAGCTTCTTCTG | GCCAAGACCTGCTTGACTG |
| DMT1 | TTGGCAATCATTGGTTCTGA | CTTCCGCAAGCCATATTTGT |
| SLC40A1 | CTACTTGGGGAGATCGGATGT | CTGGGCCACTTTAAGTCTAGC |
| FTH1 | GCCGAGAAACTGATGAAGCTGC | GCACACTCCATTGCATTCAGCC |
| FTL | ATGGGGTGCGGACTTAGAAAG | ATGGGGTGCGGACTTAGAAAG |
| NFATc1 | CACCGCATCACAGGGAAGAC | GCACAGTCAATGACGGCTC |
| NFATc2 | GAGCCGAATGCACATAAGGTC | CCAGAGAGACTAGCAAGGGG |
| NFATc3 | GCTCGACTTCAAACTCGTCTT | GATGCACAATCATCTGGCTCA |
| NFATc4 | AGAACTGGACTCAGAGGATG | ATGGAGGTGATGCGGATG |
| ACTIN | AATCGTGCGTGACATTAAGGAG | ACTGTGTTGGCGTACAGGTCTT |

**Supplementary Table 3.** Antibodies for WB, IF, IHC and IP.

| **Name** | **Application** | **Supplier** | **Cat No.** |
| --- | --- | --- | --- |
| MRP2 | WB | Cell Signaling Technology | # 4446 |
| MRP3 | WB | Cell Signaling Technology | # 39909 |
| MRP4 | WB | Cell Signaling Technology | # 12705 |
| STIM1 | WB, IP | Cell Signaling Technology | # 5668 |
| STIM1 | IHC, IF | Invitrogen | # MA1-19451 |
| SLC7A11 | WB, IP | Cell Signaling Technology | #12691 |
| SLC7A11 | IHC, IF | abcam | # ab307601 |
| 4-HNE | IHC | abcam | # ab48506 |
| GPX4 | WB | abcam | # ab125066 |
| NFATc1 | WB | Cell Signaling Technology | # 8032 |
| NFATc2 | WB, IP, IHC | Cell Signaling Technology | # 5861 |
| NFATc3 | WB | Cell Signaling Technology | # 4998 |
| NFATc4 | WB | Cell Signaling Technology | # 2183 |
| ACTIN | WB | Beyotime | AF0003 |
| TUBULIN | WB | Beyotime | AF2827 |
| Normal mouse IgG | IP | Beyotime | A7028 |
| FITC rabbit IgG | IF | Beyotime | A0562 |
| Alexa Fluor 647 mouse IgG | IF | Beyotime | A0473 |

**Supplementary Table 4.** The quality control of the raw NGS data (GSE248769).

| **Sample** | **Reads No.** | **Bases (bp)** | **Q30 (bp)** | **N (%)** | **Q20 (%)** | **Q30 (%)** |
| --- | --- | --- | --- | --- | --- | --- |
| CONTROL_n1 | 59868190 | 9040096690 | 8567896249 | 0.000751 | 98.24 | 94.78 |
| CONTROL_n2 | 52685540 | 7955516540 | 7530052750 | 0.000775 | 98.19 | 94.65 |
| CONTROL_n3 | 69658602 | 10518448902 | 9963104820 | 0.000781 | 98.22 | 94.72 |
| DR_n1 | 52572508 | 7938448708 | 7501937823 | 0.000781 | 98.14 | 94.50 |
| DR_n2 | 58213668 | 8790263868 | 8294690712 | 0.000782 | 98.07 | 94.36 |
| DR_n3 | 51042632 | 7707437432 | 7305355993 | 0.000770 | 98.25 | 94.78 |

Q30: Total number of bases with 99.9 per cent or more base identification accuracy.

N (%): Percentage of fuzzy bases.

Q20 (%): Percentage of bases with 99 per cent or more base identification accuracy.

Q30 (%): Percentage of bases with 99.9 per cent or greater base identification accuracy.

**Supplementary Table 5.** The quality control of the raw NGS data (GSE248770).

| **Sample** | **Reads No.** | **Bases (bp)** | **Q30 (bp)** | **N (%)** | **Q20 (%)** | **Q30 (%)** |
| --- | --- | --- | --- | --- | --- | --- |
| CN_n1 | 47856208 | 7226287408 | 6694636213 | 0.001948 | 97.39 | 92.64 |
| CN_n2 | 52712958 | 7959656658 | 7367725551 | 0.001862 | 97.35 | 92.56 |
| CN_n3 | 44461478 | 6713683178 | 6244678539 | 0.001958 | 97.53 | 93.01 |
| SKO_n1 | 48054064 | 7256163664 | 6702714071 | 0.002544 | 97.25 | 92.37 |
| SKO_n2 | 49254362 | 7437408662 | 6904978869 | 0.002516 | 97.45 | 92.84 |
| SKO_n3 | 39564228 | 5974198428 | 5481322329 | 0.002487 | 96.96 | 91.74 |

Q30: Total number of bases with 99.9 per cent or more base identification accuracy.

N (%): Percentage of fuzzy bases.

Q20 (%): Percentage of bases with 99 per cent or more base identification accuracy.

Q30 (%): Percentage of bases with 99.9 per cent or greater base identification accuracy.

**Supplementary Table 6.** The filtering of the raw NGS data (GSE248769).

| **Sample** | **Clean Reads No.** | **Clean Data (bp)** | **Clean Reads %** | **Clean Data %** |
| --- | --- | --- | --- | --- |
| CONTROL_n1 | 59162642 | 8918638579 | 98.82 | 98.66 |
| CONTROL_n2 | 52039984 | 7844350008 | 98.77 | 98.60 |
| CONTROL_n3 | 68816752 | 10373059008 | 98.79 | 98.62 |
| DR_n1 | 51916588 | 7827422877 | 98.75 | 98.60 |
| DR_n2 | 57451044 | 8661862390 | 98.69 | 98.54 |
| DR_n3 | 50447782 | 7605056647 | 98.83 | 98.67 |

Data were filtered by using Fastp to remove sequences with a splice at the 3' end and to remove Reads with an average mass fraction below Q20.

**Supplementary Table 7.** The filtering of the raw NGS data (GSE248770).

| **Sample** | **Clean Reads No.** | **Clean Data (bp)** | **Clean Reads %** | **Clean Data %** |
| --- | --- | --- | --- | --- |
| CN_n1 | 45255670 | 6833606170 | 94.56 | 94.56 |
| CN_n2 | 49898616 | 7534691016 | 94.66 | 94.66 |
| CN_n3 | 42058972 | 6350904772 | 94.59 | 94.59 |
| SKO_n1 | 45507976 | 6871704376 | 94.7 | 94.7 |
| SKO_n2 | 46639898 | 7042624598 | 94.69 | 94.69 |
| SKO_n3 | 37455632 | 5655800432 | 94.67 | 94.67 |

Data were filtered by using Fastp to remove sequences with a splice at the 3' end and to remove Reads with an average mass fraction below Q20.

**Supplementary Table 8.** Primers for ChIP-PCR.

| **Name** | **Sequence of  forward primer** | **Sequence of  reverse primer** | **Product** |
| --- | --- | --- | --- |
| NFAT-M1 | AACACTATAGCGCTGAGAG | GGAGATGGTGGACACAAC | -16 ~ -11 |
| NFAT-M2 | GGTGGAACGAGGAGGTGGAG | TGAAAACTCAAAGGTGTGC | -139 ~ -135 |
| NFAT-M3 | CACAAAACAGTCGCATG | GAGCAACAAAAGCTGTTC | -578 ~ -574 |
